# Feedback activation of EGFR/wild-type RAS signaling axis limits KRAS^G12D^ inhibitor efficacy in *KRAS*^*G12D*^-mutated colorectal cancer

**DOI:** 10.1038/s41388-023-02676-9

**Published:** 2023-04-05

**Authors:** Juanjuan Feng, Zhongwei Hu, Xinting Xia, Xiaogu Liu, Zhengke Lian, Hui Wang, Liren Wang, Cun Wang, Xueli Zhang, Xiufeng Pang

**Affiliations:** 1 Southern Medical University Affiliated Fengxian Hospital, The Third School of Clinical Medicine, Southern Medical University, Guangzhou, Guangdong Province 510515 China; 2 Shanghai Key Laboratory of Regulatory Biology and School of Life Sciences, East China Normal University, Shanghai, 200241 China; 3 State Key Laboratory of Oncogenes and Related Genes, Shanghai Cancer Institute, Renji Hospital, Shanghai Jiao Tong University School of Medicine, Shanghai, 200240 China

**Keywords:** Cancer therapeutic resistance, Growth factor signalling

## Abstract

Colorectal cancer (CRC), which shows a high degree of heterogeneity, is the third most deadly cancer worldwide. Mutational activation of KRAS^G12D^ occurs in approximately 10–12% of CRC cases, but the susceptibility of *KRAS*^*G12D*^-mutated CRC to the recently discovered KRAS^G12D^ inhibitor MRTX1133 has not been fully defined. Here, we report that MRTX1133 treatment caused reversible growth arrest in *KRAS*^*G12D*^-mutated CRC cells, accompanied by partial reactivation of RAS effector signaling. Through a drug-anchored synthetic lethality screen, we discovered that epidermal growth factor receptor (EGFR) inhibition was synthetic lethal with MRTX1133. Mechanistically, MRTX1133 treatment downregulated the expression of ERBB receptor feedback inhibitor 1 (ERRFI1), a crucial negative regulator of EGFR, thereby causing EGFR feedback activation. Notably, wild-type isoforms of RAS, including H-RAS and N-RAS, but not oncogenic K-RAS, mediated signaling downstream of activated EGFR, leading to RAS effector signaling rebound and reduced MRTX1133 efficacy. Blockade of activated EGFR with clinically used antibodies or kinase inhibitors suppressed the EGFR/wild-type RAS signaling axis, sensitized MRTX1133 monotherapy, and caused the regression of *KRAS*^*G12D*^-mutant CRC organoids and cell line-derived xenografts. Overall, this study uncovers feedback activation of EGFR as a prominent molecular event that restricts KRAS^G12D^ inhibitor efficacy and establishes a potential combination therapy consisting of KRAS^G12D^ and EGFR inhibitors for patients with *KRAS*^*G12D*^-mutated CRC.

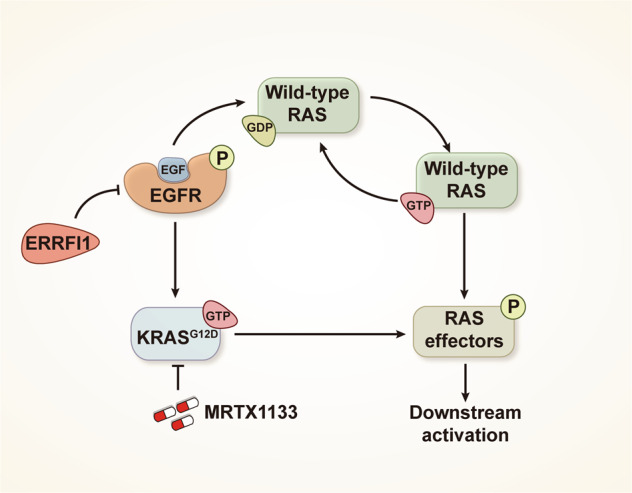

## Introduction

Colorectal cancer (CRC) is an aggressive intestinal malignancy that is currently the second most lethal cancer and the third most prevalent malignant tumor worldwide [1]. Once metastasis has occurred, the chance of 5-year survival is less than 20% [2]. Clinical treatments for CRC include laparoscopic surgery, radiotherapy, chemotherapy, and targeted therapy; however, owing to the extensive degree of heterogeneity of CRC, these treatment options still have a limited impact on the long-term survival of CRC patients [3, 4].

CRC exhibits a high degree of genetic diversity and a moderate mutational burden. Various genomic alterations mediate the initiation, progression, and metastasis of CRC, including *APC*, *TP53*, *SMAD4*, and *CTNNB1*, as well as oncogenic mitogenic signaling such as selected receptor tyrosine kinases, phosphatidylinositol 3-kinase/AKT or RAS/RAF signaling [5–7]. Notably, *KRAS* or *NRAS* mutations are present in 35–40% of CRC cases, which still lack effective targeted therapies. In particular, *KRAS*^*G12D*^ mutation is a more prevalent event, accounting for 10–12% of CRC cases [7] and is significantly associated with an increased risk of metastasis and a worse prognosis [8]. Moreover, the immune landscape of *KRAS*^*G12D*^-mutated metastatic CRC was designated as ‘immune excluded’ [9, 10], suggesting that immunotherapy will be challenging in this cancer subtype. Therefore, characterizing the biological features of *KRAS*-mutant CRC and further developing favorable treatment strategies are urgently needed and would be highly impactful.

Oncogenic KRAS mutations have been attractive and compelling drug target that have escaped decades of intense research. The approval of the covalent KRAS^G12C^-specific inhibitor sotorasib has revitalized the promise of KRAS direct targeting [11]. Clinical studies have revealed that sotorasib demonstrates satisfactory efficacy in patients with non–small cell lung cancer, with a satisfactory response rate [12, 13]. In contrast, patients with CRC have shown much lower susceptibility to the same treatment, suggesting a diverse dependency of *KRAS*-mutant cancers on specific KRAS mutant alleles [14]. This breakthrough advancement in KRAS^G12C^-specific inhibitors provides inspiration for targeting alternative KRAS mutant variants. Through a structure-based drug design strategy, MRTX1133, a high-affinity, mutation-selective, non-covalent inhibitor of KRAS^G12D^ has been discovered and reported recently [15, 16]. Preclinical studies have shown that MRTX1133 elicits potent but distinct tumor response patterns in *KRAS*^*G12D*^-mutant cancer xenograft mouse models. Specifically, the extent of MRTX1133 anti-tumor activity was less notable in CRC than in pancreatic cancer [16]. This response disparity between CRC and other *KRAS*-mutant cancer types to allele-specific inhibitors of KRAS^G12C^ and KRAS^G12D^ raises the possibility that tissue-specific molecular events may underlie the mechanism of limited efficacy, which has not yet been clearly defined.

In our study, we dissected the sensitivity of *KRAS*^*G12D*^-mutated CRC to MRTX1133 and uncovered improved treatment strategies. Through a high-throughput synthetic lethal screen, biochemical assays, and treatment experiments using *KRAS*^*G12D*^-mutant CRC cells, organoids, and xenograft models, we revealed that feedback activation of epidermal growth factor receptor (EGFR)-mediated wild-type RAS signaling contributed to the decreased effectiveness of KRAS^G12D^ inhibitor therapy in CRC. Importantly, targeting this feedback mechanism with clinically used anti-EGFR therapies, including EGFR monoclonal antibodies or kinase inhibitors, significantly increased the therapeutic efficacy of MRTX1133 against *KRAS*^*G12D*^-mutated CRC, leading to marked tumor regression and prolonged survival in mice.

## Results

### MRTX1133 provokes RAS effector signaling rebound in CRC cells

**Fig. 1:**
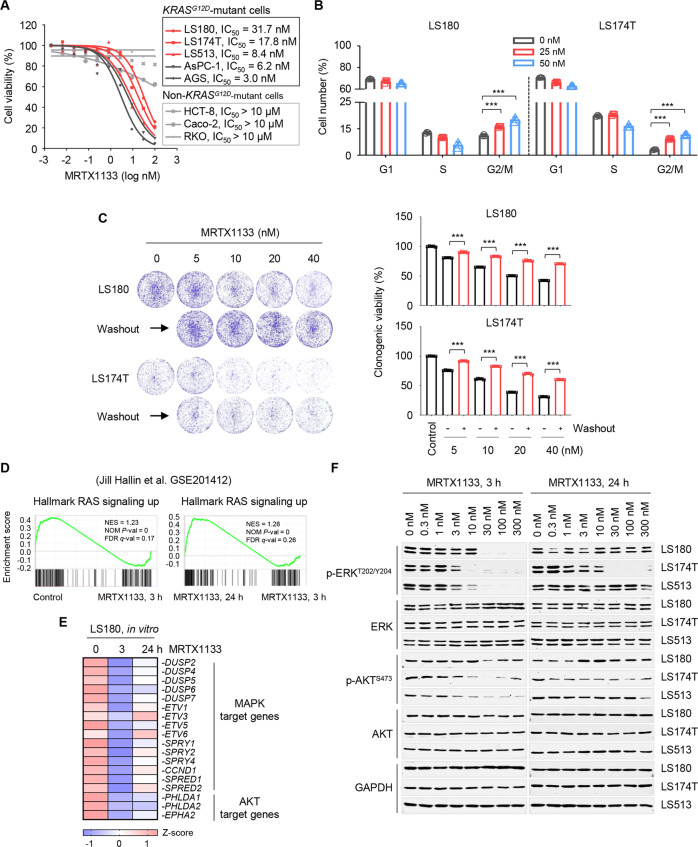
MRTX1133 provokes RAS effector signaling rebound in CRC cells. **A** Cellular activity of MRTX1133 across a panel of *KRAS*^*G12D*^ and non-*KRAS*^*G12D*^ mutant cell lines measured as the effects on 2D cell viability after a 5 days treatment. First data points of the curves represent the untreated control. Data are represented as mean ± SEM of three independent experiments with five technical replicates each. **B** Effects of MRTX1133 on cell-cycle of LS180 and LS174T cells. Cells were treated with MRTX1133 for 72 h and stained with PI to analyze the cell cycle distribution by flow cytometry. Values are expressed as the mean ± SEM of three replicates. ***P < 0.001, by one-way ANOVA with Tukey’s multiple-comparisons test. **C** Representative images (*left*) and relative numbers (*right*) of LS180 and LS174T cells treated with concentration gradients of MRTX1133 for 10 days, or various concentrations of MRTX1133 for 5 days followed by a switch to fresh medium (washout) for 5 days. Data represent the mean ± SEM of five replicates. ***P < 0.001, by one-way ANOVA with Tukey’s multiple-comparisons test. **D** Pathway enrichment was determined by GSEA from the RNA-seq dataset (Jill Hallin et al. GSE201412). LS180 cells were treated with 100 nM MRTX1133 for 3 and 24 h and then profiled by RNA sequencing (*n* = 3). NES, normalized enrichment score; NOM *P*-val, nominal *P*-value*;* FDR *q*-val, false discovery rate *q*-value. **E** Heatmap depicting differential expression of MAPK or AKT pathway-associated gene sets from the public RNA-seq dataset (Jill Hallin et al. GSE201412). *Z*-score was calculated based on counts of exon model per million mapped reads. **F** Western immunoblot analysis of ERK or AKT activation upon treatment with MRTX1133 for 3 and 24 h. GAPDH is used as loading control.

MRTX1133 is the first reported KRAS^G12D^ inhibitor with anti-tumor efficacy [16]. To test its selectivity and potency against *KRAS*^*G12D*^-mutant cancer cells, we first treated five *KRAS*^*G12D*^-mutant (LS180, LS174T, LS513, AsPC-1, and AGS) and three non-*KRAS*^*G12D*^-mutant cancer cell lines (HCT-8, Caco-2, and RKO) with MRTX1133 in a 2D cell growth assay. Our results showed that MRTX1133 impaired the growth of all *KRAS*^*G12D*^-mutant cancer cells, including three colorectal cell lines (LS180, LS174T, and LS513), with IC_50_ values ranging from 5 to 40 nM, whereas MRTX1133 had little effects on non-*KRAS*^*G12D*^-mutant cells (IC_50_ > 10 μM; Fig. 1A). These results confirmed that MRTX1133 was a highly selective KRAS^G12D^ inhibitor and elicited cytotoxicity towards *KRAS*^*G12D*^-mutated cancer cells in vitro. Furthermore, MRTX1133 treatment resulted in G2/M phase blockade in CRC cells (Fig. 1B), which is a typical consequence of KRAS mutant allele inhibition [17–19].

CRC exhibits a high degree of heterogeneity and is prone to evade various treatments, including KRAS^G12C^ inhibitor therapy [20–24]. Therefore, we suspected that *KRAS*^*G12D*^-mutated CRC cells tended to respond less to MRTX1133, even though MRTX1133 could block their growth in the nanomolar range. To test this hypothesis, we treated LS180 and LS174T cells with MRTX1133 for 5 days followed by drug withdrawal and found that cell growth resumed promptly upon MRTX1133 washout (Fig. 1C). This result suggested that MRTX1133 induced reversible growth arrest in CRC cells, which might be linked to its limited efficacy. To elucidate the molecular changes that underlay MRTX1133-induce cell cytostasis, we analyzed a published RNA sequencing (RNA-seq) dataset [16]. Gene set enrichment analysis (GSEA) demonstrated that treatment with MRTX1133 suppressed the hallmark of the RAS signaling gene signature in LS180 cells after a 3 h treatment (Fig. 1D, left), whereas this pathway was notably upregulated at the 24 h timepoint (Fig. 1D, right). In line with this, MRTX1133 decreased the expression of mitogen-activated protein kinase (MAPK) and AKT target genes as early as 3 h; however, partial recovery was observed at 24 h, indicating a rebound of the RAS effector pathway (Fig. 1E). We further ascertained these molecular events at the protein level and found that MRTX1133 transiently and dose-dependently suppressed p-ERK and p-AKT activity but lost potency after prolonged treatment (Fig. 1F). Altogether, these data indicate that feedback reactivation of RAS effector signaling could be a critical mediator responsible for reversible growth arrest and reduced efficacy elicited by MRTX1133.

### Identification of EGFR as a central component that confers MRTX1133 efficacy

**Fig. 2:**
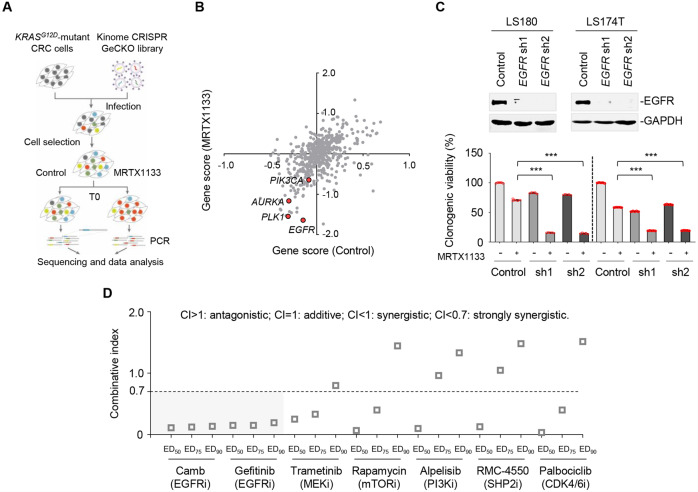
Identification of EGFR as a synthetic lethal target of MRTX1133. **A** Schematic outline of the viability-based, human kinome CRISPR-Cas9 loss-of-function screen. **B** Gene scores in untreated control versus MRTX1133-treated (25 nM) LS174T cells. The gene score was the median log_2_ fold change in the abundance of all sgRNAs targeting that gene during the culture period. Most genes, as well as non-targeting control sgRNAs, have similar scores in the presence or absence of MRTX1133. **C** Knockdown of EGFR enhance the response to MRTX1133 in LS180 and LS174T cells. *Top*, EGFR knockdown efficiency of the shRNAs was measured by immunoblot analysis. *Bottom*, Relative viability of the cultured colonies. Data are shown as mean ± SEM of five replicates. ***P < 0.001, by unpaired, two-sided Student’s *t*-test. **D** Synergistic interaction between MRTX1133 and EGFRi (cetuximab and gefitinib), MEKi (trametinib), mTORi (rapamycin), PI3Ki (alpelisib), SHP2i (RMC-4550), or CDK4/6i (albociclib) in LS174T cells. CI values at ED_50_, ED_75_, and ED_90_ were calculated using CalcuSyn software. CI values of less than 0.7 represent strongly synergism.

To identify crucial regulators that contribute to MRTX1133 effectiveness, we performed a genetic screen using the CRISPR-Cas9 library targeting the human kinome. The library consisted of 5960 single-guide RNAs (sgRNAs) targeting 503 human kinases, 10 essential genes (~10 sgRNA/gene), and 50 non-targeting gRNAs in a Cas9-expressing lentiviral vector [25]. We transduced LS174T cells with the sgRNA library, cultured them in the presence or absence of 25 nM MRTX1133, and sequenced sgRNA distribution on day 10 (Fig. 2A). The screen process did not show selection bias, as the quality and selective enrichment of sgRNA reads was comparable between the MRTX1133-treated group and the control (Supplementary Fig. S1). Our genetic screen results demonstrated that gRNAs targeting *EGFR, PLK1, AURKA, and PIK3CA* were significantly depleted in the treatment group compared to the untreated control (Fig. 2B, Supplementary Table 1). *PLK1*, *AURKA*, and *PIK3CA* have been reported as synthetic lethal genes that confer resistance to KRAS^G12C^ inhibition [26, 27]. In our genetic screen, *EGFR* was the top-ranked candidate. Considering two lines of evidence showing that: (1) EGFR is required for CRC progression and treatment response [28–30], and that (2) EGFR is the direct upstream regulator of RAS-GTPase and potentially mediates RAS effector signaling rebound that has been observed upon MRTX1133 treatment (Fig. 1), we thus focused on EGFR and further elucidated its role in endowing the effectiveness of KRAS^G12D^ inhibitor therapy. We silenced *EGFR* using short hairpin RNAs and tested whether *EGFR* loss could potentiate MRTX1133 efficacy using a clonogenic growth assay (Fig. 2C). Our results demonstrated that *EGFR* knockdown significantly augmented the MRTX1133 response, producing a marked inhibition of clonogenic growth in LS180 and LS174T cells.

To verify the results of the kinome-centered genetic screen, we additionally conducted a mini-screen using chemical inhibitors specifically targeting EGFR, MEK, mTOR, phosphoinositide 3-kinase (PI3K), SHP2, or CDK4/6, which are key factors associated with drug tolerance in CRC [31–34]. We assessed the antigrowth effects of various drug pairs consisting of MRTX1133 and chemical inhibitors in LS174T cells by calculating the combination index (CI) at 50%, 75%, and 90% of the effective dose (ED_50_, ED_75_, and ED_90_) for each drug pair. Our results showed that MEK, mTOR, PI3K, SHP2, and CDK4/6 inhibitors exhibited modest and less consistent combinatorial effects with MRTX1133 (Fig. 2D). In contrast, concurrent treatment with EGFR inhibitors (cetuximab or gefitinib) and MRTX1133 produced a strong synergy, with CI values at ED_50_, ED_75_, and ED_90_ all less than 0.7 (Fig. 2D). The results of our two independent screens collectively revealed that EGFR inhibition was synthetically lethal with MRTX1133, substantiating the central role of EGFR in conferring MRTX1133 efficacy.

### ERRFI1 downregulation by MRTX1133 causes EGFR reactivation

**Fig. 3:**
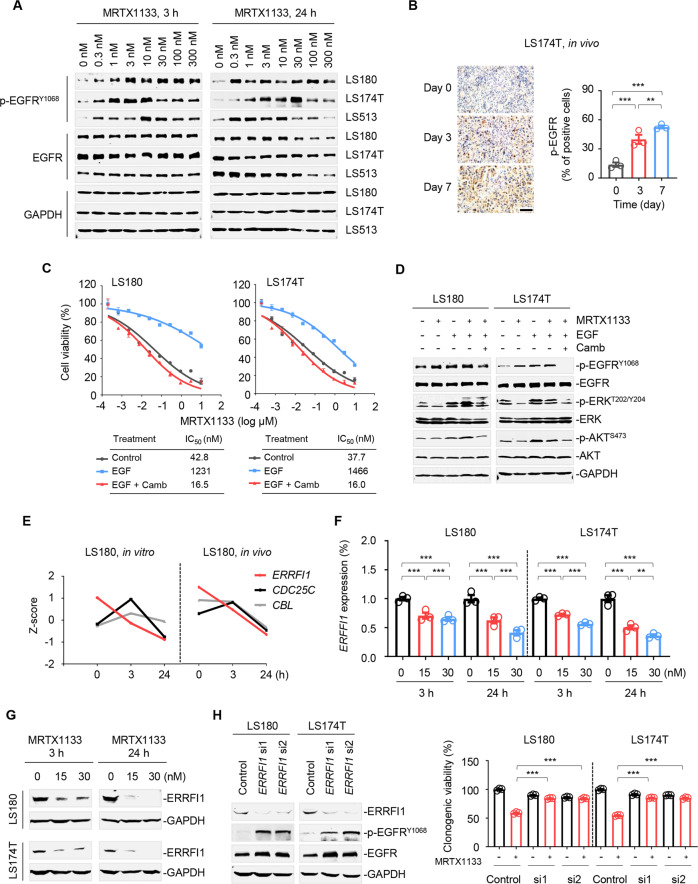
ERRFI1 downregulation by MRTX1133 causes EGFR adaptive reactivation. **A** Effects of MRTX1133 on the p-EGFR expression in *KRAS*^*G12D*^-mutant CRC cells. LS180, LS174T, and LS513 cells were treated with indicated concentration of MRTX1133 for 3 or 24 h. Cell lysates were probed with antibodies against p-EGFR, EGFR and GAPDH. **B** Immunohistochemical analysis of p-EGFR in LS174T xenograft tumors. Tumors were isolated after 3 or 7-day administration of MRTX1133. Immunohistochemical staining assay were detected (*left*). Scale bars, 200 μm. Percent of tumor cell positive for p-EGFR assessed (*right*). Data represent the mean ± SEM. **P < 0.01, ***P < 0.001, by one-way ANOVA with Tukey’s multiple-comparisons test. **C** Cell viability assay. LS180 and LS174T cells were treated with EGF (20 ng/mL) and/or cetuximab (50 μg/mL), or with untreated control, and detected their sensitivity to MRTX1133. Each point on the dose-response curves represents five technical replicates. Data are shown as mean ± SEM. The charts below show the IC_50_ values. **D** Western immunoblot analysis of LS180 and LS174T cells treated with EGF (20 ng/mL), cetuximab (50 μg/mL), and/or MRTX1133 (30 nM) as indicated for 30 min. **E** RNA-seq analysis of **E**GFR negative regulators (*ERRFI1*, *CDC25C*, and *CBL*) in vitro and in vivo (Jill Hallin et al. GSE201412). **F**, **G** MRTX1133 downregulated *ERRFI1* expression at the transcription (**F**) and protein levels (**G**) in a concentration- or time-dependent manner. LS180 and LS174T cells were treated with MRTX1133 for the indicated durations. ERRFI1 expression was detected by western immunoblot and RT-qPCR assays. Data are presented as mean ± SEM of three technical replicates. **P < 0.01, and ***P < 0.001, by one-way ANOVA with Tukey’s multiple-comparisons test. **H** *ERFFI1* silencing impaired the inhibitory effects of MRTX1133 on LS180 and LS174T cells. *Left*, *ERRFI1* knockdown efficiency in LS180 and LS174T cells by immunoblot analysis. *Right*, colony formation assay. Data represent the mean ± SEM of biological triplicates. ***P < 0.001, by one-way ANOVA with Tukey’s multiple-comparisons test.

The contribution of EGFR to RAS signaling outputs and the close link between EGFR signaling and CRC [14, 28] prompted us to evaluate EGFR status in the presence of MRTX1133. LS180, LS174T, and LS513 cells were treated with increasing concentrations of MRTX1133, and EGFR activation was detected using an antibody against EGFR p-Y1068. We observed that MRTX1133 dose-dependently increased p-EGFR expression upon a 3 h drug exposure (Fig. 3A, *left*). Activated EGFR lasted for at least 24 h (Fig. 3A, *right*), indicating that EGFR activation was an early and durable response. Indeed, EGFR feedback activation accorded with the rebound of RAS effector signaling in MRTX1133-treated cells (Fig. 1G). To verify EGFR reactivation in vivo, we treated LS174T xenograft tumors with 20 mg/kg MRTX1133 every 12 h for 3 and 7 days. Immunohistochemistry data demonstrated that p-EGFR levels were significantly elevated in treated tumor xenografts (Fig. 3B). Importantly, enhanced EGFR signaling by adding exogenous EGF to the medium was sufficient to decrease the sensitivity of LS180 and LS174T cells to MRTX1133, an effect that could be further blocked by cetuximab combination therapy (Fig. 3C). Furthermore, EGF supplementation prevented the MRTX1133-mediated decline in p-ERK and p-AKT levels, whereas the addition of cetuximab could diminish this effect (Fig. 3D). Collectively, these data highlight the role of EGFR reactivation in endowing MRTX1133 resistance in *KRAS*^*G12D*^-mutant CRC cells.

To gain further insight into how EGFR signaling is activated in response to MRTX1133, we analyzed RNA-seq profiles of treated CRC cells and xenograft tumors [16]. We examined transcriptional changes in genes encoding intrinsic negative regulators of EGFR, including *ERRFI1* [29, 35, 36], *CDC25C* [37], and *CBL* [38] (Fig. 3E). Notably, only *ERRFI1* mRNA expression decreased in a time-dependent manner, both in vitro and in vivo, unlike the inconsistent expression patterns of *CDC25C* and *CBL* (Fig. 3E). Given that ERRFI1 is associated with tumor development and drug resistance [29, 39, 40], we further explored the functional role of ERRFI1 in EGFR feedback activation in the context of MRTX1133. Our results showed a notable decrease in both ERRFI1 mRNA and protein expression in MRTX1133-treated LS180 and LS174T cells (Fig. 3F, G). Moreover, siRNA-mediated knockdown of ERRFI1 increased EGFR activation and conferred MRTX1133 sensitivity (Fig. 3H). Overall, these data indicate that ERRFI1 downregulation by MRTX1133 resulted in EGFR adaptive activation and MRTX1133 insensitivity.

### EGFR-mediated wild-type RAS reactivation bypasses KRAS^G12D^

**Fig. 4:**
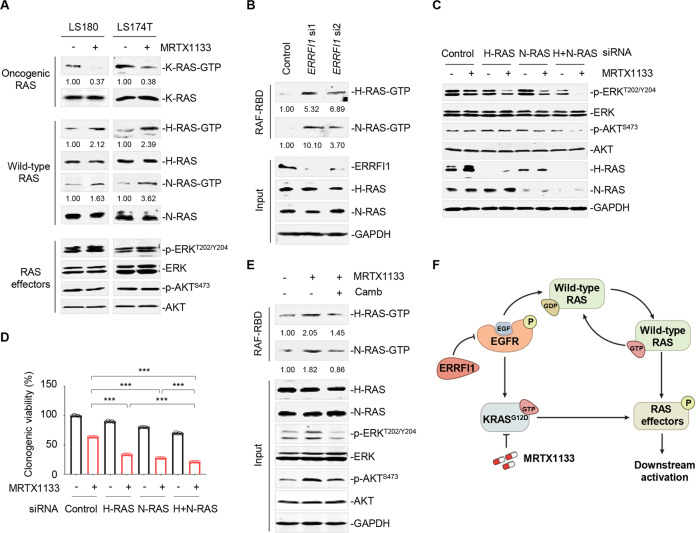
Wild-type RAS drives RAS effector signaling rebound. **A** MRTX1133 increased wild-type RAS-GTP levels. LS180 and LS174T cells were treated with MRTX1133 for 24 h and lysates were subjected to GST-RAF-RBD pulldown assay. Immunoblot analysis was subsequently performed. The relative density of K-RAS-, H-RAS-, and N-RAS-GTP bands versus their respective RAS inputs is shown. **B** *ERRFI1* silencing increased wild-type RAS-GTP levels. Lysates of LS180 cells after *ERRFI1* silencing were subjected to GST-RAF-RBD pulldown assay. The relative density of RAS-GTP bands versus their respective RAS inputs was quantified. **C** Wild-type RAS knockdown impaired MRTX1133-mediated RAS signaling activation. LS180 cells were subject to siRNA knockdown of H-RAS, N-RAS, or both wild-type RAS and treated with or without MRTX1133 for 24 h. Immunoblot analysis was performed for p-ERK and p-AKT. **D** Wild-type RAS silencing potentiated MRTX1133 efficacy in LS180 cells. Cells were subject to siRNA knockdown of H-RAS, N-RAS, or both wild-type RAS and treated with 30 nM MRTX1133 for 7 days. Data are presented as mean ± SEM of three technical replicates. ***P < 0.001, by one-way ANOVA with Tukey’s multiple-comparisons test. **E** Increased wild-type RAS-GTP levels require EGFR activation. LS180 cells were treated with 30 nM MRTX1133, 50 μg/mL cetuximab or their combination for 24 h. H-RAS and N-RAS were detected by western immunoblot assay. The relative density of GTP-bound forms of RAS proteins is shown. **F** A proposed model for the regulation of EGFR/wild-type RAS signaling by MRTX1133.

Previous studies have shown that oncogenic RAS regulates the basal effector pathway, whereas wild-type RAS mediates signaling downstream of the activated receptor tyrosine kinases [41]. Next, we sought to characterize the downstream components of EGFR to decipher the role of wild-type RAS in the presence of MRTX1133. We treated LS180 and LS174T cells with MRTX1133 for 24 h (the time point at which MRTX1133 provoked RAS effector signaling rebound) and performed isoform-specific RAS-GTP binding (RBD) pull-down assays to determine the expression levels of RAS active isoforms. Our results showed that MRTX1133, a high-affinity, mutation-selective inhibitor of KRAS^G12D^, effectively suppressed oncogenic RAS-GTP (K-RAS-GTP) levels in LS180 and LS174 cells (Fig. 4A). In contrast, the levels of active GTP-bound isoforms of wild-type RAS, including H-RAS and N-RAS, apparently increased after a 24-h treatment (Fig. 4A). As a result, a rebound in RAS effector signaling was observed simultaneously. Since ERRFI1 downregulation by MRTX1133 contributed to EGFR reactivation, we wondered whether ERRFI1 also linked to EGFR-mediated wild-type RAS reactivation. Our results demonstrated a notable increase in active GTP-bound forms of both wild-type H-RAS and N-RAS in LS180 cells after transient knockdown of *ERRFI1*, indicating that wild-type RAS reactivation was also dependent on ERRFI1 regulation (Fig. 4B).

Next, we assessed whether feedback activation of H-RAS and N-RAS played a functional role in the adaptive reactivation of RAS signaling. We interfered with H-RAS and N-RAS expression using small interfering RNAs and measured the degree of RAS signaling activation in treated cells. Our results showed that transient knockdown of H-RAS, N-RAS, or both wild-type RAS proteins was sufficient to abrogate the rebound in p-ERK and p-AKT levels induced by MRTX1133 (Fig. 4C). The greatest reduction in RAS effector signaling was observed with the loss of both wild-type RAS proteins. Accordingly, loss of both RAS proteins elicited a greater sensitizing effect on MRTX1133 as compared to knockdown each alone (Fig. 4D). Altogether, these data establish that wild-type isoforms of RAS, but not oncogenic RAS, contributed to RAS effector signaling rebound in the context of MRTX1133.

Next, we tested whether blocking EGFR activity was capable of decreasing EGFR-mediated feedback signaling into wild-type RAS. Our results showed that EGFR inhibition by cetuximab blocked the increase in H-RAS, N-RAS, and RAS effector activity in MRTX1133-treated cells (Fig. 4E). These findings collectively support the hypothesis that signals upstream of EGFR provoked wild-type RAS and RAS effector signaling rebound in the context of KRAS^G12D^ inhibition (Fig. 4F).

### EGFR inhibition sensitizes MRTX1133 treatment in vitro

**Fig. 5:**
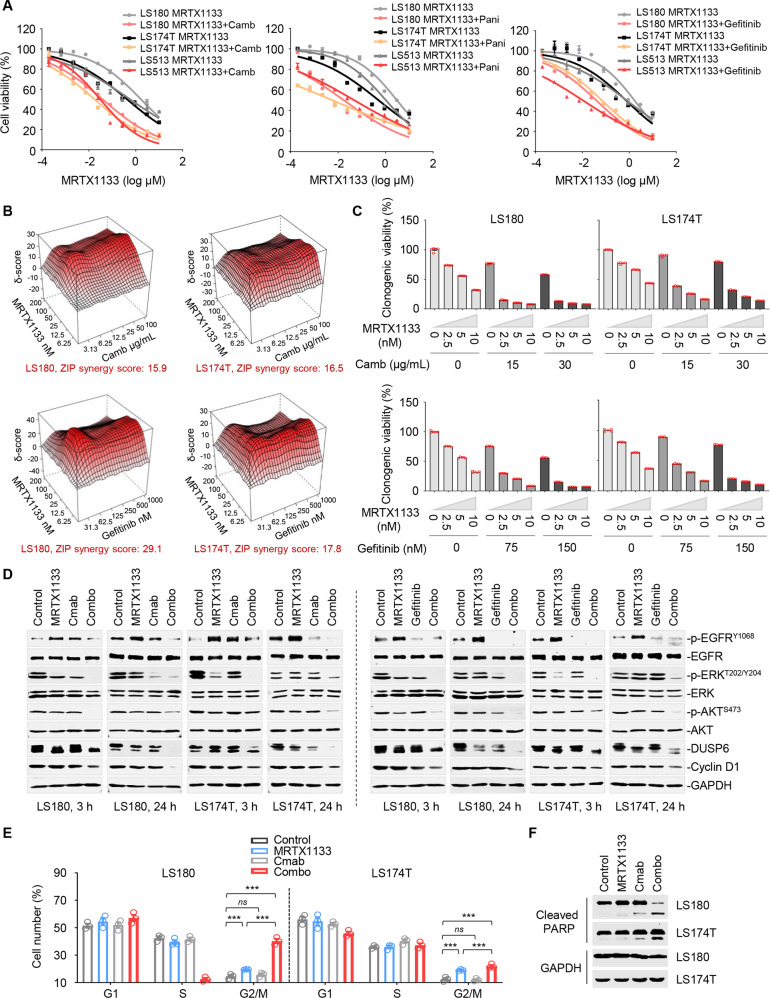
Targeted inhibition of EGFR synergizes with MRTX1133 in vitro. **A** EGFR inhibition enhanced the efficacy of MRTX1133 in *KRAS*^*G12D*^-mutant CRC cell lines. LS180, LS174T and LS513 cells were treated with concentration gradients of MRTX1133 with or without cetuximab (50 μg/mL), panitinib (50 μg/mL), or gefitinib (150 nM) for 5 days. Shown are cell viability curves. Data represent the mean ± SEM of biological triplicates. **B** Synergy diagram of MRTX1133 and cetuximab (*top*), MRTX1133 and gefitinib (*bottom*) analyzed by R package “synergyfinder”. LS180 and LS174T cells were treated for 72 h with various concentrations of the indicated inhibitors. The concentrations of EGFRi or MRTX1133 were used in a two-fold dilution series (3.13, 6.25, 12.5, 25, 50, and 100 μg/mL for cetuximab; 31.3, 62.5, 125, 250, 500, and 1000 nM for gefitinib; 6.25, 12.5, 25, 50, 100, and 200 nM for MRTX1133). Relative cell viability was subsequently measured. ZIP values were simulated using zero interaction potency model analysis. **C** Inhibition of clonogenic viability by the combined regimen. LS180 and LS174T cells were treated with MRTX1133, EGFRi (cetuximab and gefitinib), or their combination as indicated. Quantified clonogenic viability inhibition results are shown. Data are mean ± SEM of five technical replicates. **D** LS180 and LS174T cells treated with the indicated MRTX1133, EGFRi (cetuximab and gefitinib), or their combination for 3 or 24 h were assessed by western immunoblot. **E** Cell-cycle profiles. Data represent the mean ± SEM of biological triplicates. ***P < 0.001; *ns*, no significant, by one-way ANOVA with Tukey’s multiple-comparisons test. **F** Immunoblot analysis of cleaved PARP in LS180 and LS174T cells. Cells were treated with MRTX1133, EGFRi (cetuximab and gefitinib), or their combination for 72 h. Cell lysates were probed with antibodies against cleaved PARP and GAPDH.

Given that KRAS^G12D^ inhibition reactivated EGFR/wild-type RAS signaling, we sought to test whether the current EGFR-specific monoclonal antibodies (cetuximab and panitumumab) [6] or kinase inhibitors (gefitinib) [42] were capable of eliminating reactivated RAS effector signaling and sensitizing *KRAS*^*G12D*^-mutant CRC cells to KRAS^G12D^ inhibitor treatment. To this end, we first applied a fixed concentration of cetuximab, panitumumab, or gefitinib, along with increasing concentrations of MRTX1133, and then examined their antigrowth effects. Our results showed that EGFR inhibition effectively sensitized *KRAS*^*G12D*^-mutant CRC cells to MRTX1133 treatment (Fig. 5A). Next, we examined the synergistic interaction between EGFR inhibitors and MRTX1133 using a short-term proliferation assay. Our results showed a notable synergy for each drug pair, with zero interaction potency (ZIP) synergy scores ranging from 15 to 30 (a score greater than 1 indicates a synergistic interaction) (Fig. 5B, Supplementary Fig. S2A). Cooperative effects of MRTX1133 and EGFR inhibitors were also observed in a long-term colony formation assay (Fig. 5C, Supplementary Fig. S2B). In accordance with this, western immunoblot analysis revealed that concomitant targeting of KRAS^G12D^ and EGFR led to sustained inhibition of p-ERK and p-AKT as well as ERK downstream targets, DUSP6 and cyclin D1 (Fig. 5D, Supplementary Fig. S2C). Moreover, the combination of MRTX1133 and cetuximab induced a strengthened G2/M blockade (Fig. 5E) and pronounced apoptosis in treated cells (Fig. 5F). Overall, these data indicate that EGFR inhibition effectively augments MRTX1133 therapeutic efficacy in vitro.

### Co-treatment of MRTX1133 and EGFR inhibitors impedes CRC organoid growth

**Fig. 6:**
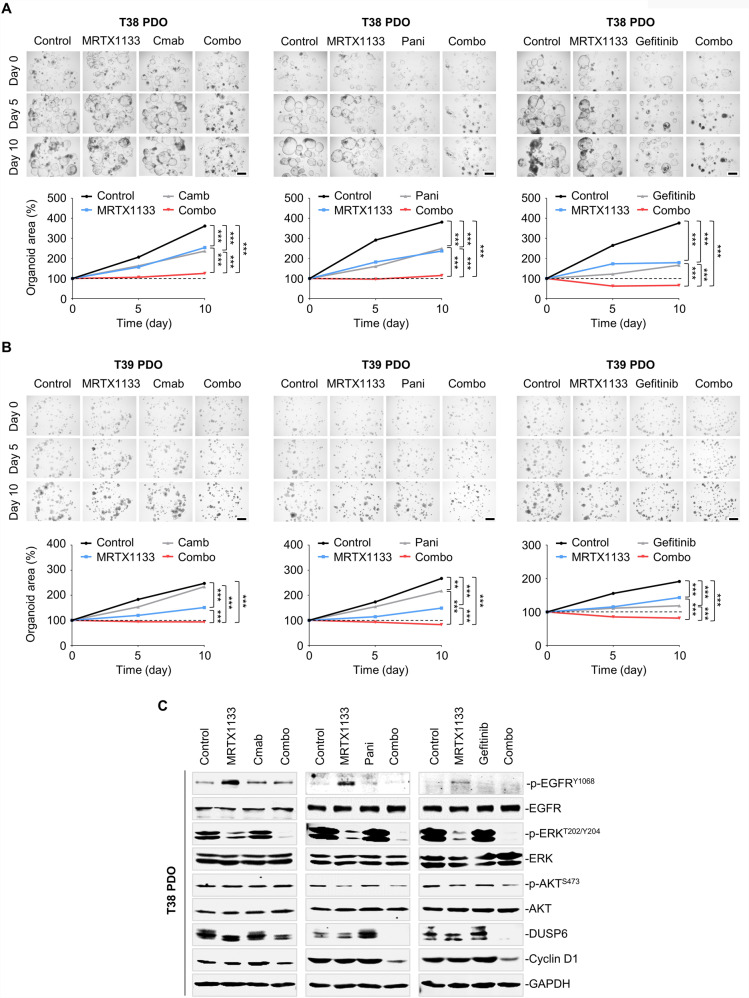
Co-treatment of MRTX1133 and EGFR inhibitors impedes CRC organoid growth. **A**, **B** Inhibition of organoids proliferation by the combined regimen. T38 (**A**) and T39 (**B**) PDOs were treated with various concentrations of the indicated inhibitors. *Top*, Representative bright-field microscopy images of organoids. *Bottom*, Quantification of organoid size change curves after drug combinations. Data represent the mean ± SEM. **P < 0.01, and ***P < 0.001, by one-way ANOVA with Tukey’s multiple-comparisons test. **C** T38 PDO were treated with MRTX1133 and EGFRi for 24 h, and western immunoblot analysis was performed with the indicated antibodies. GAPDH served as a loading control.

Patient-derived organoids (PDOs) retain the molecular features of the tumors from which they are derived. Importantly, the ex vivo responses of PDOs to clinically relevant treatments correlate with the responses observed in patient tumors [43]. To test whether and to what extent our findings were applicable to more clinically relevant models, we established two *KRAS*^*G12D*^-mutant PDO models (named T38 and T39) and further tested the therapeutic effects of MRTX1133, EGFR inhibitors (cetuximab, panitumumab, and gefitinib), or their combinations. Although the single agents MRTX1133, cetuximab, panitumumab, or gefitinib inhibited the growth of T38 PDO, the combination of MRTX1133 and EGFR inhibition elicited more potent antigrowth activity and restricted organoid size to the baseline (Fig. 6A). Similar results were observed for the T39 PDO (Fig. 6B). Moreover, molecular studies demonstrated that the combination treatment led to a more notable shutdown of ERK and AKT activity than single-agent treatment (Fig. 6C). In accordance with the p-ERK suppression, the abundance of DUSP6 and cyclin D1 decreased simultaneously. In T38 PDO models, we found that AKT activity was less affected by co-treatment when compared to ERK, suggesting that MAPK signaling might play a more important role in the combined efficacy. Overall, these data suggest that dual targeting of KRAS^G12D^ and EGFR effectively abrogates the proliferation of CRC organoids by blocking the RAS effector signaling.

### EGFR inhibition improves the therapeutic efficacy of MRTX1133 in vivo

**Fig. 7:**
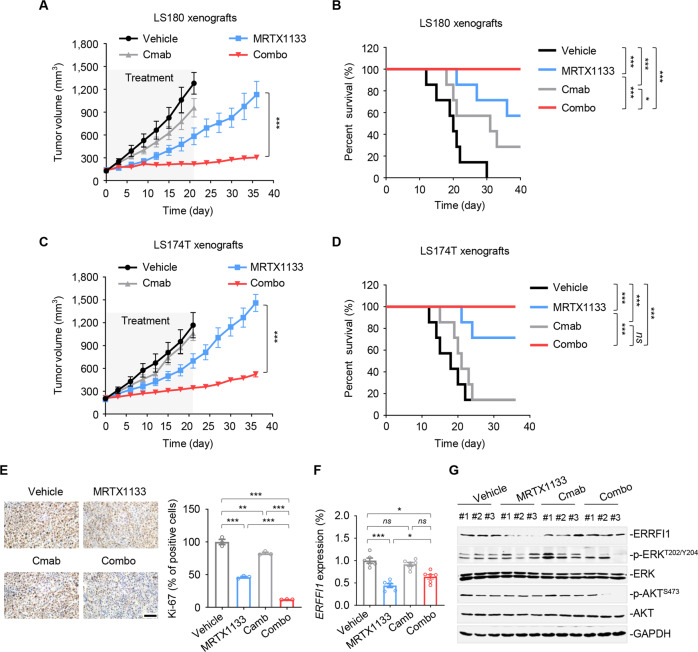
EGFR inhibition sensitizes *KRAS*^*G12D*^-mutant CRC to MRTX1133 in vivo. **A** Growth curves of LS180 xenografts in mice (*n* = 7 per group). Athymic nude mice bearing LS180 xenografts were treated with vehicle, MRTX1133 (20 mg/kg, intraperitoneally once a day), cetuximab (50 mg/kg, intraperitoneally twice a week), or combination for 21 days. Tumor volumes were measured every three days. Data represent the mean ± SEM. ***P < 0.001, by unpaired, two-sided Student’s *t*-test. **B** Kaplan-Meier survival curves of LS180 xenografts after the indicated treatment (*n* = 7 per group). *P < 0.05, and ***P < 0.001, by log-rank tests. **C** Growth curves of LS174T xenografts in mice (*n* = 7 per group). Athymic nude mice bearing LS174T xenografts were treated as in (**A**). Data represent the mean ± SEM. ***P < 0.001, by unpaired, two-sided Student’s *t*-test. **D** Kaplan-Meier survival curves of LS174T xenografts (*n* = 7 per group). ***P < 0.001; *ns*, not sig*n*ificant, by log-rank tests. **E** Immunohistochemical staining for Ki-67 from LS174T xenografts tumors. Representative images of immunohistochemical staining for Ki-67 in tumor samples after 21-day treatment (*left*). Scale bars, 200 μm. Percent of tumor cell positive for Ki-67 assessed (*right*). Data represent the mean ± SEM. **P < 0.01, and ***P < 0.001, by one-way ANOVA with Tukey’s multiple-comparisons test. **F** RT-qPCR analysis of *ERRFI1* in LS174T xenograft tumors. At the end of the treatment, LS174T xenograft tumors were isolated from mice. RT-qPCR assay were detected. Data represent the mean ± SEM of six independent biologically samples. *P < 0.05, and *** *P* < 0.001; *ns*, no significant, by one-way ANOVA with Tukey’s multiple-comparisons test. **G** Immunoblot analysis of p-ERK, p-AKT, and ERRFI1 in LS174T xenograft tumors. Three biologically independent samples per group are shown.

Next, we tested the cooperative activity of KRAS^G12D^ and EGFR inhibitors using *KRAS*^*G12D*^-mutated CRC cell xenograft mouse models. Mice bearing LS180 xenografts were treated with vehicle, MRTX1133, cetuximab, or a combination of both, for 21 days. MRTX1133 or cetuximab alone minimally suppressed xenograft tumor growth within the treatment period, whereas their combination demonstrated robust and synergistic anti-tumor activity, suggesting a sensitizing effect of cetuximab on MRTX1133 (Fig. 7A). We also examined tumor growth after treatment withdrawal. Our results demonstrated that tumors in the drug combination group grew substantially slower than those in the MRTX1133 monotherapy group after therapy termination (Fig. 7A), highlighting the durable efficacy of the combined regimen. As a surrogate of mouse survival, animals were counted as dead when the tumors reached 1000 mm^3^. In fact, cetuximab at the tested dose was ineffective for mouse survival because agents that block EGFR hardly benefit CRC patients whose tumors harbor KRAS mutations. In contrast to cetuximab, MRTX1133 alone or in combination with cetuximab prolonged mouse survival (Fig. 7B).

This in vivo treatment efficacy was also observed in LS174T xenograft tumors. Consistently, MRTX1133 and cetuximab combination therapy significantly retarded the growth of LS174T xenografts even after drug withdrawal and markedly prolonged mouse survival compared to each single agent (Fig. 7C, D). Impressively, the addition of cetuximab to MRTX1133 was well tolerated, as weight loss and systematic toxicity were within acceptable limits in both xenograft mouse models during the course of treatment (Supplementary Tables 3 and 4). Immunohistochemical analysis demonstrated that co-treatment with MRTX1133 and cetuximab significantly reduced Ki67 expression in LS174T xenograft tumors (Fig. 7E). In agreement with in vitro observations, ERRFI1 mRNA and protein expression were significantly decreased by MRTX1133 treatment, whereas cetuximab addition could rescue this effect (Fig. 7F, G). Moreover, the combination of MRTX1133 and cetuximab inhibited cancer cell-specific p-ERK and p-AKT levels (Fig. 7G). Collectively, these in vivo data suggest potent and sustained anti-tumor activity of the combined therapy of KRAS^G12D^ and EGFR inhibitors.

## Discussion

CRC is a genetically heterogeneous disease with various oncogenic alterations, leaving more opportunities and opening the door for targeted therapy. Among the accumulated genetic alterations, the KRAS^G12D^ mutation is the most prevalent, accounting for 10–12% of CRC cases [44]. In our study, we uncovered a mechanistic explanation for why *KRAS*^*G12D*^-mutated CRC cells responded less to KRAS^G12D^ inhibitor therapy. Through drug-anchored genetic and chemical screens, biochemical analysis, and therapeutic studies, we found that signaling feedback via the EGFR pathway served as the dominant mechanism for the intrinsic resistance of CRC cells to KRAS^G12D^ inhibitor therapy. Notably, wild-type RAS isoforms, rather than oncogenic RAS, mediate signaling downstream of the activated EGFR provoked by MRTX1133. We found that EGFR inhibition was synthetically lethal with KRAS^G12D^ blockade, as co-targeting KRAS^G12D^ and EGFR resulted in more potent cell apoptosis in vitro and tumor shrinkage in vivo (Fig. 7). These data support the exploration of dual KRAS^G12D^/EGFR inhibition as a strategy to improve treatment response and extend the clinical benefits to patients with KRAS^G12D^-mutated colorectal tumors.

The small GTPase KRAS is mutated and constitutively active in approximately one in seven of all human tumors [45]. Mutational activation of KRAS is frequently observed in pancreatic cancer, CRC, and non–small cell lung cancer. In addition to distinct signaling properties linked to each mutant form of KRAS, genetic studies have revealed that specific mutant KRAS alleles are associated with distinct tissue-specific genetic dependencies [46], highlighting the complexity that drives the divergent clinical outcomes of KRAS allele-specific inhibitors. For example, patients with KRAS^G12C^-mutated non–small cell lung cancer achieved a durable clinical benefit from sotorasib, the first approved KRAS^G12C^ mutant-selective inhibitor, whereas patients with CRC bearing the same mutation responded less to the same treatment [12]. Therefore, investigating the distinct roles of KRAS mutant alleles in the context of the tissue of origin is crucial to developing effective treatment strategies for KRAS-dependent cancers and to create tissue-specific applicability of KRAS inhibitors. Recently, the first potent and selective non-covalent KRAS^G12D^ allele-specific inhibitor, MRTX1133, was developed [15, 16]. According to preclinical studies, CRC responds less to MRTX1133 than pancreatic cancer in tumor xenograft mouse models. This may be because genomic instability or a hypermutated background constrains dependency on KRAS in CRC. Several selected genes such *EGFR* and *PIK3CA* have been shown to modify the response to MRTX1133. In agreement with a recently published study showing the synergism between MTRX1133 and EGFR inhibitors [16], we also found that the signaling feedback activation of EGFR is a major mediator of intrinsic resistance to KRAS^G12D^ blockade through a synthetic lethality screen. We further identified that MRTX1133 promoted EGFR reactivation through downregulating ERRFI1, a crucial inhibitor of EGFR (Fig. 3E–G). It has been reported that ERRFI1 not only blocks EGFR’s activity by clamping onto its kinase domain to rapidly decrease EGFR signaling [47], but also directs EGFR to the lysosomes for digestion for a longer-lasting inhibitory effect [48]. In line with this two-tiered model in which ERRFI1 regulates EGFR, ERRFI1 genetic silencing increased both phosphorylated and total EGFR abundance as well as EGFR-mediated wild-type RAS reactivation in *KRAS*^*G12D*^-mutant CRC cells (Fig. 3H and Fig. 4B). EGFR reactivation has also been reported to restrict the efficacy of KRAS^G12C^ inhibition in KRAS^G12C^-mutated CRC [14]. These independent studies collectively suggest that CRC cells are primed to depend on EGFR signaling to evade KRAS-targeted therapy in a lineage-specific manner. Given that colorectal tumors are more addicted to EGFR signaling for growth and survival than other types of malignancies, the conclusion that EGFR feedback signaling confers partial resistance to KRAS^G12C^ and KRAS^G12D^ inhibition therapy is likely a universal mechanism for other KRAS variant oncoprotein inhibitors in CRC. Agents that block EGFR benefit many CRC patients, with the exception of those whose tumors harbor KRAS mutations. Crucially, as a consequence of this EGFR feedback model, combining oncogenic RAS inhibitors with clinically used EGFR inhibitors could lead to increased tumor regression and extended survival in patients with KRAS-mutant CRC.

Although feedback activation of EGFR signaling has been established as a putative event elicited by targeted inhibition of mitogenic pathways [14, 29], its downstream signaling in the context of KRAS allele-specific inhibitors, such as MRTX1133, is still an enigma. In our study, we found that the activity of wild-type RAS isoforms, including H-RAS and N-RAS, markedly increased upon MRTX1133-mediated oncogenic RAS blockade (Fig. 4A), indicating that oncogenic and wild-type RAS may play divergent but complementary roles in response to mitogenic signaling perturbations. Moreover, pharmacological inhibition of EGFR suppressed the reactivation of wild-type RAS in the presence of MRTX1133, supporting the notion that wild-type RAS isoforms mediate signaling downstream of EGFR (Fig. 4E). It has been revealed that oncogenic RAS intrinsically desensitizes signal transduction originating from EGFR. Therefore, genetic depletion of oncogenic RAS with siRNA oligonucleotides relieves this negative feedback, leading to hyperactivation of EGFR and wild-type RAS signaling [41]. In accordance with this knowledge, the pharmacological depression of oncogenic RAS with MRTX1133 also caused signaling rebound of EGFR and wild-type RAS in our study, further demonstrating the relief of the negative-feedback control (Figs. 3, 4). Recent work also suggested the importance of feedback reactivation of the MAPK pathway through wild-type H-RAS and N-RAS [49]. In the activated state, RAS protein interacts with downstream effectors to mediate signaling pathways crucial for cellular proliferation and survival [50, 51]. In our study, we discovered that the rebound wild-type RAS isoforms predominantly activated downstream p-ERK and p-AKT, as the co-treatment of MRTX1133 and EGFR inhibitors led to potent inhibition of ERK and AKT in both *KRAS*^*G12D*^-mutated CRC cells and organoids (Figs. 5, 6).

In summary, our findings elucidate the mechanism by which feedback activation of the EGFR/wild-type RAS axis limits the therapeutic effects of KRAS^G12D^ inhibitor therapy in CRC patients. Our study also establishes the therapeutic potential of the concurrent blockade of KRAS^G12D^ and EGFR as an improved treatment for patients with *KRAS*^*G12D*^-mutated colorectal tumors.

## Materials and Methods

### Cell lines and chemicals

LS174T, LS513, AsPC-1, AGS, HCT-8, Caco-2, and RKO cell lines were purchased from the Chinese Academy of Sciences (Shanghai, China), and LS180 cell line was obtained from Mingzhou Biotechnology (Ningbo, China). LS174T cells were cultured in Dulbecco’s modified Eagle’s medium (DMEM) supplemented with 10% fetal bovine serum (FBS; Thermo Fisher Scientific, Waltham, MA, USA) and 1% antibiotic mixture containing 100 U/mL penicillin and 100 mg/mL streptomycin (P/S). LS513, AsPC-1, and HCT-8 cells were cultured in Roswell Park Memorial Institute (RPMI)-1640 medium, supplemented with 10% FBS and 1% P/S. AGS cells were maintained in Ham’s F-12K medium containing 10% fetal bovine serum (FBS) and 1% P/S. Caco-2 cells were cultured in Minimum Essential Medium (MEM) supplemented with 20% FBS, 1% P/S, 1% sodium pyruvate, 0.1 mM non-essential amino acids, and 2 mM _L_-glutamine. RKO and LS180 cells were maintained in MEM containing 20% FBS and 1% P/S. All cell lines were determined to be *Mycoplasma* free using a Myco-Lumi^TM^ kit (C0297; Beyotime, Shanghai, China) and tested using short tandem repeats.

The antineoplastic agents used in this study, including trametinib (S2673, MEK inhibitor), rapamycin (S1039, mTOR inhibitor), alpelisib (S2814, PI3K inhibitor), RMC-4550 (S8718, SHP2 inhibitor), palbociclib (S4482, CDK4/6 inhibitor), MRTX1133 (E1051, KRAS^G12D^ inhibitor), gefitinib (S1025, EGFR kinase inhibitor), cetuximab (A2000, EGFR monoclonal antibody) and panitumumab (A2018, EGFR monoclonal antibody) were purchased from Selleck (Shanghai, China).

### Patient-derived organoid culture

The use of human tumor tissue followed the guidelines approved by the local ethics committee of hospitals or institutions. Written informed consent was obtained from all the patients. T39 and T38 PDOs were obtained from two distinct patients with KRAS^G12D^ CRC who underwent surgical resection at the Southern Medical University Affiliated Fengxian Hospital (Shanghai, China). PDOs were established as previously described [52]. Briefly, CRC tissues was cleaned in cold PBS with P/S and then minced into tiny fragments in a sterile dish on ice. Tissue fragments were then subjected to enzymatic digestion in 10 mL digestion medium containing 9 mL DMEM, 500 U/mL collagenase IV (C9407, Sigma Aldrich, St. Louis, USA), 1.5 mg/mL collagenase II (C8150, Solarbio, Beijing, China), and 0.1 mg/mL dispase type II (D4693, Sigma Aldrich) on an orbital shaker at 37 °C for 20–40 minutes. The cell suspension was filtered using a 70 μm cell strainer to remove large fragments. The derived single-cell suspension was centrifuged at 400 × g for 5 min and resuspended in an organoid culture medium. Tumor cells were seeded into Matrigel in a pre-warmed 12-well plate and overlaid with 500 µL organoid culture medium. The medium was replaced every 2–3 days. The culture medium was supplemented with 10 µM of the ROCK inhibitor Y27632 (S6390; Selleck) for the first 2 days.

The organoid culture medium was composed of Advanced DMEM/F12 medium containing 100 U/mL P/S, 10 mM HEPES, 2 mM Glutamine, 50 mg/mL R-spondin-1 (11083-HNAS, Sino Biological, Houston, USA), 100 ng/mL Noggin (50688-M02H, Sino Biological), 1 × B27 supplement (17504-044, Invitrogen, Waltham, USA), 1.25 mM N-acetyl-cysteine (A9165, Sigma Aldrich), 10 mM Nicotinamide (N0636, Sigma Aldrich), 10 nM Gastrin (G9145, Sigma Aldrich), 50 ng/mL recombinant human EGF (50482-MNCH, Sino Biological), 500 nM A83-01 (S7692, Selleck), 3 µM SB202190 (S1077, Selleck), 10 nM Prostaglandin E2 (P6532, Sigma Aldrich) and 100 μg/ml Primocin (ant-pm-1, Invivogen, Hongkong, China).

### Organoids preparation and drug tests

Organoids in good condition were harvested, passaged, and seeded into 48-well cell culture plates as described above. The organoid density was adjusted to 5–10/µL of Matrigel before seeding. Then, 300 µL of the organoid culture medium was added to each well. For drug tests, the organoid culture medium was removed, replaced with 300 µL of drug-containing culture medium, and refreshed every three days. Organoids were photographed every five days during drug treatment. The size of the living organoids was measured using the ImageJ software (NIH, Bethesda, USA).

### CRISPR-Cas9 functional genetic screen

Synthetic lethal screening and data analyses were performed as previously described [25]. Briefly, LS174T cells were transfected with lentivirus expressing the CRISPR library that targets the human kinome. The infection was carried out at an MOI of 0.5 with a 500-fold coverage. After puromycin (2 µg/mL) selection for seven days, the surviving cells were harvested. A portion of the cells (equaling a 500-fold coverage of the library) was collected for gDNA extraction (T0), while the remaining cells were plated in 15 cm dishes and cultured in the presence or absence of 25 nM MRTX1133. Cells were refreshed with fresh media or METX1133-containing media every three days and passaged when they were confluent. Ten days after drug treatment, the cells were harvested, and gDNA was extracted using the Blood and Cell Culture DNA Maxi kit (69506, Qiagen) according to the manufacturer’s protocols. The kinome CRISPR library prepared from the genomic DNA was subjected to HiSeq analysis. The CRISPR viability score was calculated as the mean of the Log_2_-transformed fold change of all sgRNAs.

### Western immunoblot

Cell lysates were harvested using a radioimmunoprecipitation assay lysis buffer supplemented with protease and phosphatase inhibitors. The total protein concentration of the cell lysates was determined using a BCA protein quantification kit (20201ES76; Yeasen, Shanghai, China) according to the manufacturer’s protocol. Proteins from each sample (10–50 µg) were separated on 8% or 12% sodium dodecyl sulfate-polyacrylamide gel electrophoresis gels and transferred to nitrocellulose membranes (Millipore, Billerica, MA, USA). After blocking in Tris-buffered saline with Tween (TBST) buffer containing 5% w/v bovine serum albumin, the membranes were probed with primary antibodies overnight at 4 °C. The membranes were washed thrice in TBST and incubated with secondary antibodies for 1 h at room temperature. Image acquisition and band intensity quantification were performed using an Odyssey infrared imaging system (LI-COR Biosciences, LincoIn, USA) and Image J software (NIH, Bethesda, USA), respectively.

The following primary antibodies were obtained from Cell Signaling Technology and used at a dilution of 1:1000: anti-ERRFI1 (#2440), anti-EGFR (#4267), anti-phospho-EGFR^Y1068^ (#3777), anti-ERK1/2 (#9102), anti-phospho-ERK1/2 (#4370), anti-AKT (#9272), anti-phospho-AKT^S473^ (#4060), and anti-PARP (#9542). Anti-H-RAS (sc-35) and anti-N-RAS (sc-31) antibodies were purchased from Santa Cruz Biotechnology and used at 1:200 dilution. Anti-cyclin D1 (CY5404), anti-DUSP6 (CY5420), and anti-GAPDH (AB0037) were obtained from Abcam (Cambridge, UK) and used at a 1:1000 dilution. Anti-K-RAS (ab275876) was purchased from Abcam and used at a 1:1000 dilution.

### RAS activity assay

RAS activity was assessed using a GST-RAF-RBD pulldown assay kit (17-10393 Millipore), followed by immunoblotting with RAS isoform-specific antibodies. Briefly, cells were lysed in 1% TX100-TNM lysis buffer (20 mM Tris-HCl [pH 7.5], 5 mM MgCl_2_, 150 mM NaCl, and 1% Triton X-100) supplemented with 1 mM DTT, protease, and phosphatase inhibitors. Cell lysates (500 µg) were loaded into the columns, together with 10 µL of packed GST-RAF-RBD beads, and rotated for 2 h at 4 °C. The beads were then washed thrice with 1 mL of cold lysis buffer and boiled in SDS sample buffer. Pulldown and total lysates were subjected to western blotting as described above.

### Immunohistochemical staining

Tumor samples were obtained after drug treatment, fixed in 4% paraformaldehyde solution, embedded in paraffin, and cut into 4 µm sections. Tissue specimens were routinely deparaffinized, rehydrated, subjected to antigen retrieval, and incubated in 3% hydrogen peroxide to block endogenous peroxidases. Subsequently, the tissue specimens were blocked with 3% bovine serum albumin and incubated with primary antibodies anti-phospho-EGFR^Y1068^ (1:200, #3777, Cell Signaling Technology) and anti-Ki67 (1:100, #9449, Cell Signaling Technology) at 4 °C overnight, followed by detection with biotin-conjugated secondary antibody and avidin peroxidase, and visualization using aminoethyl carbazole chromogen. Images were obtained using a Leica microscope (DM4000b, Leica). Three to five fields per independent tumor from each treatment condition were used for quantification.

### Quantitative PCR analysis

Reverse transcription quantitative PCR (RT-qPCR) was performed as previously described [53]. The primer sets used are listed in Supplementary Table 2. The relative copy number was determined by calculating the fold-change difference in the gene of interest relative to *β*-actin.

### shRNA transfection

To prepare retroviral particles, 293 T cells were plated in a 10 cm dish and shRNAs targeting EGFR were transfected with Lipo8000™ Transfection reagent (C0533, Beyotime). For cell infection, LS174T and LS180 cells were plated at a density of 2 × 10^6^ cells per 6 cm dish and infected with the virus from 293 T cells 48 h after transfection. Stable shRNA-mediated EGFR knockdown cells were generated after selection using puromycin (2 µg/mL). All lentiviral shRNA vectors were retrieved from an arrayed TRC human genome-wide shRNA collection. Western blotting was used to assess the efficiency of shRNA-mediated gene knockdown. The shRNA targeting sequences are shown in Supplementary Table 2.

### siRNA transfection

Gene knockdown was performed by RNA interference. Briefly, the cells were transfected with 200 pM siRNA for 48 h. The cells were either treated with the appropriate agents for the cell proliferation assay or lysed for western blot analysis to determine knockdown efficiency. The siRNA sequences used are listed in Supplementary Table 2.

### Cell viability assay

Cells were seeded in 96-well plates at a density of 2000–4000 cells per well and allowed to adhere overnight. The cells were treated with various concentrations of the indicated drugs for five days. Cell viability was determined using the CellTiter 96 cell proliferation assay kit (G3580; Promega, Madison, WI, USA), according to the manufacturer’s instructions. IC_50_ values were determined by GraphPad Prism 9 using a 3-parameter dose-response model. For drug synergy analysis, cells were treated with a single agent or a fixed-ratio combination for 5 days. CI values were calculated using CalcuSyn software, version 2 (Reachsoft, China), and ZIP values were simulated using zero interaction potency model analysis (http://synergyfinder).

### Colony formation assay

The cells were seeded in 12-well plates. After overnight incubation, the cells were treated with various inhibitors for 10 days (the inhibitor-containing medium was replaced every three days). At the end of the treatment period, the colonies were fixed and stained. Crystal violet was removed from the colonies using 10% acetic acid and the absorbance was measured at 595 nm. The relative cell viability was calculated by setting the untreated group to 100%.

### Cell cycle analysis

Cells were grown in 6 cm dishes and treated with the indicated drugs or drug combinations for 72 h. Cells were fixed and stained with propidium iodide (Sigma-Aldrich). The cell cycle was analyzed using flow cytometry (FACSCalibur, BD, USA).

### Animal studies

All animal experiments were approved by East China Normal University and performed in accordance with the guidelines of the Institutional Animal Care and Use Committee. Mice were euthanized once the tumor volume reached 1500 mm^3^. For *KRAS*^*G12D*^-mutated colorectal tumor xenograft models, 5 × 10^6^ CRC cells (LS180 and LS174T) were suspended in a 1:1 mixture of PBS and Matrigel before implantation by direct subcutaneous injection into the flanks of 6–8-week-old nude mice. Once the tumor volume reached 75–150 mm^3^, tumor-bearing mice were treated daily with vehicle (i.p., twice daily, 10% DMSO, 40% PEG300, 5% Tween-80, and 45% saline), MRTX1133 (20 mg/kg, i.p., twice daily, dissolved in 10% DMSO, 40% PEG300, 5% Tween-80, and 45% saline), cetuximab (50 mg/kg, i.p.), or a combination of MRTX1133 and cetuximab. Tumor volume was measured every three days using the following formula: tumor volume (mm^3^) = (length × width^2^) × 0.52. For survival curve analysis, treatment was prolonged until the tumor reached a total volume of 1500 mm^3^.

### Statistical analysis

Data are presented as the mean ± SEM, unless otherwise stated. Statistical tests were performed using Microsoft Excel and GraphPad Prism (version 7.0). Two-tailed unpaired *t*-test were used to compare two groups. For the comparison of multiple groups, one-way ANOVA with Tukey’s multiple comparison test was used. The log-rank test was used for the survival analysis. Statistical significance was set at *P < 0.05, **P < 0.01, and ***P < 0.001. The other specific tests are described in the figure legends.

## Supplementary information

Supplementary information

## Data Availability

RNA-seq data were obtained from the Gene Expression Omnibus with accession number GSE201412.

## References

[1] Sung H, Ferlay J, Siegel RL, Laversanne M, Soerjomataram I, Jemal A (2021). Global Cancer Statistics 2020: GLOBOCAN Estimates of Incidence and Mortality Worldwide for 36 Cancers in 185 Countries. CA Cancer J Clin.

[2] Bien J, Lin A (2021). A review of the diagnosis and treatment of metastatic colorectal cancer. JAMA.

[3] Kuipers EJ, Grady WM, Lieberman D, Seufferlein T, Sung JJ, Boelens PG (2015). Colorectal cancer. Nat Rev Dis Primers.

[4] Keum N, Giovannucci E (2019). Global burden of colorectal cancer: Emerging trends, risk factors and prevention strategies. Nat Rev Gastroenterol Hepatol.

[5] Cancer Genome Atlas N. (2012). Comprehensive molecular characterization of human colon and rectal cancer. Nature.

[6] Xie YH, Chen YX, Fang JY (2020). Comprehensive review of targeted therapy for colorectal cancer. Signal Transduct Target Ther.

[7] Yaeger R, Chatila WK, Lipsyc MD, Hechtman JF, Cercek A, Sanchez-Vega F (2018). Clinical sequencing defines the genomic landscape of metastatic colorectal cancer. Cancer Cell.

[8] Boutin AT, Liao WT, Wang M, Hwang SS, Karpinets TV, Cheung H (2017). Oncogenic Kras drives invasion and maintains metastases in colorectal cancer. Genes Dev.

[9] Picard E, Verschoor CP, Ma GW, Pawelec G (2020). Relationships between immune landscapes, genetic subtypes and responses to immunotherapy in colorectal cancer. Front Immunol.

[10] Ciardiello D, Vitiello PP, Cardone C, Martini G, Troiani T, Martinelli E (2019). Immunotherapy of colorectal cancer: Challenges for therapeutic efficacy. Cancer Treat Rev.

[11] Canon J, Rex K, Saiki AY, Mohr C, Cooke K, Bagal D (2019). The clinical KRAS(G12C) inhibitor AMG 510 drives anti-tumour immunity. Nature.

[12] Skoulidis F, Li BT, Dy GK, Price TJ, Falchook GS, Wolf J (2021). Sotorasib for lung cancers with KRAS p.G12C Mutation. N Engl J Med.

[13] Tsai YS, Woodcock MG, Azam SH, Thorne LB, Kanchi KL, Parker JS (2022). Rapid idiosyncratic mechanisms of clinical resistance to KRAS G12C inhibition. J Clin Invest.

[14] Amodio V, Yaeger R, Arcella P, Cancelliere C, Lamba S, Lorenzato A (2020). EGFR blockade reverts resistance to KRAS(G12C) inhibition in colorectal cancer. Cancer Discov.

[15] Wang X, Allen S, Blake JF, Bowcut V, Briere DM, Calinisan A (2022). Identification of MRTX1133, a Noncovalent, Potent, and Selective KRAS(G12D) Inhibitor. J Med Chem.

[16] Hallin J, Bowcut V, Calinisan A, Briere DM, Hargis L, Engstrom LD (2022). Anti-tumor efficacy of a potent and selective non-covalent KRAS(G12D) inhibitor. Nat Med.

[17] Li C, Zhao N, An L, Dai Z, Chen X, Yang F (2021). Apoptosis-inducing activity of synthetic hydrocarbon-stapled peptides in H358 cancer cells expressing KRAS(G12C). Acta Pharm Sin B.

[18] Zhang Z, Miao L, Lv C, Sun H, Wei S, Wang B (2013). Wentilactone B induces G2/M phase arrest and apoptosis via the Ras/Raf/MAPK signaling pathway in human hepatoma SMMC-7721 cells. Cell Death Dis.

[19] Xie C, Li Y, Li LL, Fan XX, Wang YW, Wei CL (2017). Identification of a new potent inhibitor targeting KRAS in non-small cell lung cancer cells. Front Pharmacol.

[20] Corcoran RB, Andre T, Atreya CE, Schellens JHM, Yoshino T, Bendell JC (2018). Combined BRAF, EGFR, and MEK Inhibition in Patients with BRAF(V600E)-Mutant Colorectal Cancer. Cancer Discov.

[21] Kopetz S, Desai J, Chan E, Hecht JR, O’Dwyer PJ, Maru D (2015). Phase II pilot study of vemurafenib in patients with metastatic BRAF-mutated colorectal cancer. J Clin Oncol.

[22] Linnekamp JF, Wang X, Medema JP, Vermeulen L (2015). Colorectal cancer heterogeneity and targeted therapy: a case for molecular disease subtypes. Cancer Res.

[23] Fakih MG, Kopetz S, Kuboki Y, Kim TW, Munster PN, Krauss JC (2022). Sotorasib for previously treated colorectal cancers with KRAS(G12C) mutation (CodeBreaK100): A prespecified analysis of a single-arm, phase 2 trial. Lancet Oncol.

[24] Awad MM, Liu S, Rybkin II, Arbour KC, Dilly J, Zhu VW (2021). Acquired Resistance to KRAS(G12C) Inhibition in Cancer. N Engl J Med.

[25] Jin H, Shi Y, Lv Y, Yuan S, Ramirez CFA, Lieftink C (2021). EGFR activation limits the response of liver cancer to lenvatinib. Nature.

[26] Xue JY, Zhao Y, Aronowitz J, Mai TT, Vides A, Qeriqi B (2020). Rapid non-uniform adaptation to conformation-specific KRAS(G12C) inhibition. Nature.

[27] Misale S, Fatherree JP, Cortez E, Li C, Bilton S, Timonina D (2019). KRAS G12C NSCLC models are sensitive to direct targeting of KRAS in combination with PI3K inhibition. Clin Cancer Res.

[28] Corcoran RB, Ebi H, Turke AB, Coffee EM, Nishino M, Cogdill AP (2012). EGFR-mediated re-activation of MAPK signaling contributes to insensitivity of BRAF mutant colorectal cancers to RAF inhibition with vemurafenib. Cancer Discov.

[29] Wu Q, Zhen Y, Shi L, Vu P, Greninger P, Adil R (2022). EGFR Inhibition Potentiates FGFR Inhibitor Therapy and Overcomes Resistance in FGFR2 Fusion-Positive Cholangiocarcinoma. Cancer Discov.

[30] Martinelli E, Ciardiello D, Martini G, Troiani T, Cardone C, Vitiello PP (2020). Implementing anti-epidermal growth factor receptor (EGFR) therapy in metastatic colorectal cancer: challenges and future perspectives. Ann Oncol.

[31] Di Nicolantonio F, Vitiello PP, Marsoni S, Siena S, Tabernero J, Trusolino L (2021). Precision oncology in metastatic colorectal cancer - from biology to medicine. Nat Rev Clin Oncol.

[32] Zhao M, Scott S, Evans KW, Yuca E, Saridogan T, Zheng X (2021). Combining Neratinib with CDK4/6, mTOR, and MEK Inhibitors in Models of HER2-positive Cancer. Clin Cancer Res.

[33] Sorokin AV, Kanikarla Marie P, Bitner L, Syed M, Woods M, Manyam G (2022). Targeting RAS Mutant Colorectal Cancer with Dual Inhibition of MEK and CDK4/6. Cancer Res.

[34] Liu C, Lu H, Wang H, Loo A, Zhang X, Yang G (2021). Combinations with Allosteric SHP2 Inhibitor TNO155 to Block Receptor Tyrosine Kinase Signaling. Clin Cancer Res.

[35] Maity TK, Venugopalan A, Linnoila I, Cultraro CM, Giannakou A, Nemati R (2015). Loss of MIG6 Accelerates Initiation and Progression of Mutant Epidermal Growth Factor Receptor-Driven Lung Adenocarcinoma. Cancer Discov.

[36] Descot A, Hoffmann R, Shaposhnikov D, Reschke M, Ullrich A, Posern G (2009). Negative regulation of the EGFR-MAPK cascade by actin-MAL-mediated Mig6/Errfi-1 induction. Mol Cell.

[37] Prahallad A, Sun C, Huang S, Di Nicolantonio F, Salazar R, Zecchin D (2012). Unresponsiveness of colon cancer to BRAF(V600E) inhibition through feedback activation of EGFR. Nature.

[38] Jiang R, Tang J, Chen Y, Deng L, Ji J, Xie Y (2017). The long noncoding RNA lnc-EGFR stimulates T-regulatory cells differentiation thus promoting hepatocellular carcinoma immune evasion. Nat Commun.

[39] Endo H, Okami J, Okuyama H, Nishizawa Y, Imamura F, Inoue M (2017). The induction of MIG6 under hypoxic conditions is critical for dormancy in primary cultured lung cancer cells with activating EGFR mutations. Oncogene.

[40] Yoo JY, Kim TH, Shin JH, Marquardt RM, Muller U, Fazleabas AT (2022). Loss of MIG-6 results in endometrial progesterone resistance via ERBB2. Nat Commun.

[41] Young A, Lou D, McCormick F (2013). Oncogenic and wild-type Ras play divergent roles in the regulation of mitogen-activated protein kinase signaling. Cancer Discov.

[42] Cooper AJ, Sequist LV, Lin JJ (2022). Third-generation EGFR and ALK inhibitors: Mechanisms of resistance and management. Nat Rev Clin Oncol.

[43] Drost J, Clevers H (2018). Organoids in cancer research. Nat Rev Cancer.

[44] Li S, Balmain A, Counter CM (2018). A model for RAS mutation patterns in cancers: finding the sweet spot. Nat Rev Cancer.

[45] Prior IA, Hood FE, Hartley JL (2020). The Frequency of Ras Mutations in Cancer. Cancer Res.

[46] Cook JH, Melloni GEM, Gulhan DC, Park PJ, Haigis KM (2021). The origins and genetic interactions of KRAS mutations are allele- and tissue-specific. Nat Commun.

[47] Zhang X, Pickin KA, Bose R, Jura N, Cole PA, Kuriyan J (2007). Inhibition of the EGF receptor by binding of MIG6 to an activating kinase domain interface. Nature.

[48] Frosi Y, Anastasi S, Ballaro C, Varsano G, Castellani L, Maspero E (2010). A two-tiered mechanism of EGFR inhibition by RALT/MIG6 via kinase suppression and receptor degradation. J Cell Biol.

[49] Ryan MB, Coker O, Sorokin A, Fella K, Barnes H, Wong E (2022). KRAS(G12C)-independent feedback activation of wild-type RAS constrains KRAS(G12C) inhibitor efficacy. Cell Rep.

[50] Thein KZ, Biter AB, Hong DS (2021). Therapeutics targeting mutant KRAS. Annu Rev Med.

[51] Punekar SR, Velcheti V, Neel BG, Wong KK (2022). The current state of the art and future trends in RAS-targeted cancer therapies. Nat Rev Clin Oncol.

[52] Yao Y, Xu X, Yang L, Zhu J, Wan J, Shen L (2020). Patient-derived organoids predict chemoradiation responses of locally advanced rectal cancer. Cell Stem Cell.

[53] Guo J, Liu Y, Lv J, Zou B, Chen Z, Li K (2021). BCL6 confers KRAS-mutant non-small-cell lung cancer resistance to BET inhibitors. J Clin Invest.

