# Supplementary material for: Feedback activation of EGFR/wild-type RAS signaling axis limits KRASG12D inhibitor efficacy in KRASG12D-mutated colorectal cancer
Source: Oncogene. 2023 Apr 5;42(20):1620–33. doi: 10.1038/s41388-023-02676-9 (PMC10181928; doi:10.1038/s41388-023-02676-9)
Supplement: Supplementary file 1 — Supplementary information [file 41388_2023_2676_MOESM1_ESM.pdf]

This supplementary Information contains

1. **Supplementary Figure 1.** Scatter plots of sgRNA representation (log_2 number of reads) between with or without drug treatment in LS174T cells.
2. **Supplementary Figure 2.** EGFR inhibition sensitizes *KRAS*^{G12D}-mutant CRC to MRTX1133.
3. **Supplementary Tables.**

Supplementary Figure 1

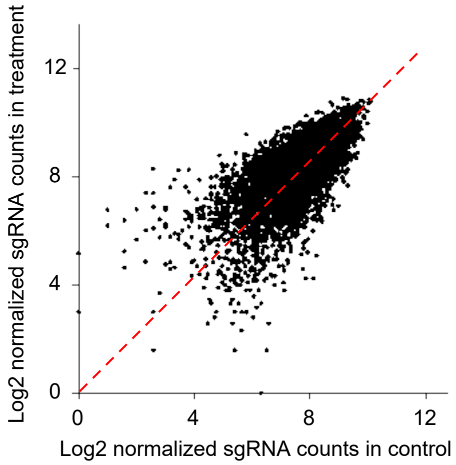

Supplementary Figure 1 | Scatter plots of sgRNA representation (log_2 number of reads) between with or without drug treatment in LS174T cells.

Supplementary Figure 1

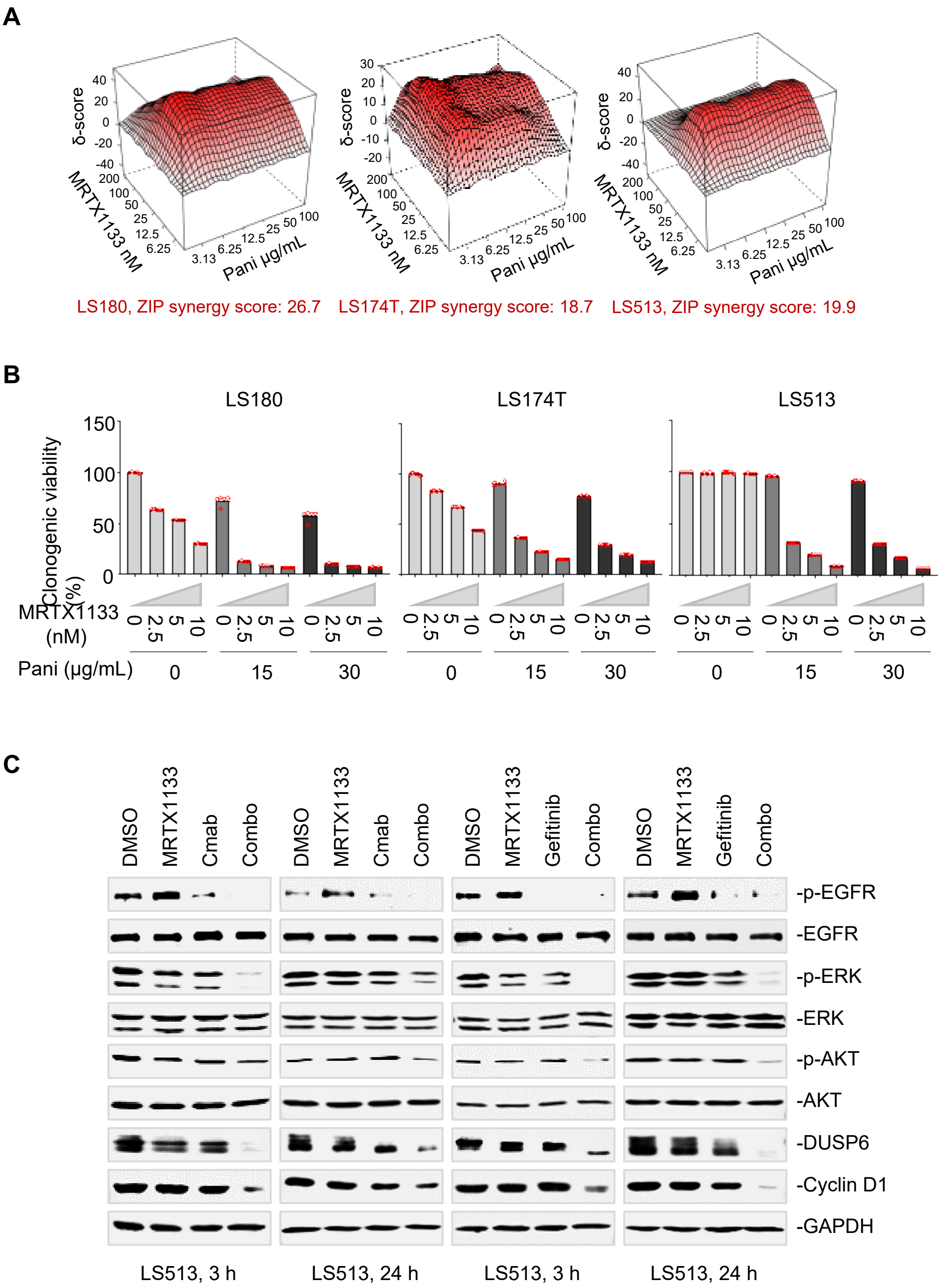

Supplementary Figure 2 | EGFR inhibition sensitizes *KRAS*^{G12D}-mutant CRC to MRTX1133. **(A)** Synergy diagram of MRTX1133 and EGFRi analyzed by R package “synergyfinder”. LS180, LS174T, and LS513 cells were treated for 72 h with various concentrations of the indicated inhibitors. Relative cell viability was subsequently measured. ZIP values were simulated using zero interaction potency model analysis. **(B)** Inhibition of clonogenic viability by the combined regimen. LS180, LS174T, and LS513 cells were treated with MRTX1133, panitumumab, or their combination as indicated. Quantified clonogenic viability inhibition results are shown. Data are mean ± SEM of five technical replicates. **(C)** LS513 cells treated with the indicated MRTX1133, EGFRi (cetuximab and gefitinib), or their combination for 3 or 24 h were assessed by western immunoblot.

Table S1. Gene scores of the MRTX1133 screen

Gene name	Median sgRNA score (Control)	Median sgRNA score (MRTX1133)
AAK1	0.260906993	-0.05663107
AATK	0.211704128	0.114585633
ABL1	0.033240645	-0.043190994
ABL2	0.047329967	-0.209086706
ACVR1	0.136105013	-0.13718136
ACVR1B	0.019601177	0.248174451
ACVR1C	-0.008236617	-0.125008331
ACVR2A	-0.175700924	-0.385873747
ACVR2B	0.161987171	0.287060893
ACVRL1	0.55338316	-0.022308353
ADCK1	0.027963857	-0.28165174
ADCK2	0.304642227	0.157134029
ADCK3	0.144581266	0.042412842
ADCK4	0.118766509	0.243012307
ADCK5	-0.080889808	-0.124693357
ADRBK1	0.214574104	0.275225457
ADRBK2	0.031583737	0.459863548
AKT1	0.024990063	0.047058366
AKT2	-0.119718864	-0.307838131
AKT3	-0.32445994	-0.17925324
ALK	0.201647628	0.318417466
ALPK1	-0.01693503	0.257224878
ALPK2	0.027593468	-0.118461127
ALPK3	0.073097273	0.027281932
AMHR2	0.01908286	0.314724959
ANKK1	0.538424128	0.64512388
ARAF	0.067029043	0.302718238
ATM	0.080650536	-0.230011217
ATR	0.438335362	0.310647566
AURKA	-0.300361283	-1.163145093
AURKB	0.091381443	-0.478605052
AURKC	-0.06238924	0.220923783
AXL	-0.034464953	0.030074214
BCKDK	-0.012223593	-0.077530865
BLK	-0.011949724	0.014683438
BMP2K	0.601006205	0.568035042
BMPR1A	-0.251214852	-0.84585536
BMPR1B	0.100757408	0.296626299
BMPR2	0.072097826	-1.733341506
BMX	-0.14419179	0.246197733
BRAF	-0.116169976	-0.294107806
BRSK1	-0.137011816	-0.411760629
BRSK2	0.073031822	0.10402926
BTK	0.005111496	-0.656835585
BUB1	-0.607483553	-1.100878389
BUB1B	-0.147665727	-1.049153615
C9orf96	0.264523756	0.41285824
CAMK1	-0.055386327	-0.125962002
CAMK1D	-0.161256762	-0.663809536
CAMK1G	-0.113158138	0.667348777
CAMK2A	-0.155551691	-0.104689484
CAMK2B	-0.101769639	-0.791222132
CAMK2D	-0.159188746	-0.214888076
CAMK2G	-0.123212367	-0.076739596
CAMK4	0.137548793	0.30270456
CAMKK1	0.390199152	0.507183635
CAMKK2	0.120860834	0.097770331
CAMKV	-0.017911099	0.281419678
CASK	0.142806939	0.251451353
CDC42BPA	-0.061877131	-0.38789685
CDC42BPB	0.045509905	-0.530878165

CDC42BPG	-0.237155064	-0.913247657
CDC7	-0.34212639	-1.025950313
CDK1	-0.366466061	-0.792523212
CDK10	-0.04559513	-0.51319341
CDK11B	-0.031143755	-0.250690614
CDK12	0.139286124	0.158889266
CDK13	0.53694602	0.643401734
CDK14	0.181994717	0.377229372
CDK15	0.111692755	0.29709818
CDK16	0.128688072	0.07293139
CDK17	-0.02748457	-0.210861249
CDK18	0.138901741	0.142535156
CDK19	0.153368893	0.305203637
CDK2	-0.205568953	-0.07034463
CDK20	0.213533656	0.107804334
CDK3	-0.060689338	-0.054568716
CDK4	0.593477063	-0.249232199
CDK5	-0.019421782	-0.321886104
CDK6	-0.16024499	-0.769772893
CDK7	-0.293243851	-1.427981474
CDK8	-0.090219724	0.13115367
CDK9	-0.228506514	-0.618541237
CDKL1	0.215721963	0.482041764
CDKL2	-0.278773561	-0.072229788
CDKL3	-0.077774341	-0.196522923
CDKL4	0.187436496	0.019602466
CDKL5	-0.049597369	-0.094881117
CHEK1	-0.330960275	-1.524595779
CHEK2	0.13845358	0.61326602
CHUK	-0.072224834	-0.269362965
CIT	-0.185576116	-0.432313429
CLK1	0.071983305	-0.220789634
CLK2	-0.174690233	-0.255442297
CLK3	0.251344716	0.131465054
CLK4	0.048134634	0.295134139
COPB1	-0.154970924	-0.266214295
CSF1R	0.120101661	0.152386008
CSK	0.255286008	-0.010944567
CSNK1A1	-0.124581955	-0.585417405
CSNK1A1L	0.112019074	-0.203574299
CSNK1D	0.112801693	0.023286301
CSNK1E	-0.025510053	0.487052118
CSNK1G1	0.002470685	0.134589437
CSNK1G2	0.028567624	-0.310932996
CSNK1G3	-0.131799723	-0.69458704
CSNK2A1	-0.153343606	-0.198856337
CSNK2A2	-0.201129798	-0.418955567
CSNK2A3	-0.496951684	-0.040270127
DAPK1	0.169536462	0.10483494
DAPK2	0.045864348	-0.048463415
DAPK3	0.228870685	0.36154172
DCLK1	-0.011682358	-0.069353302
DCLK2	-0.083610018	-0.123591611
DCLK3	-0.41202466	-0.016257701
DDR1	-0.131928731	-0.027791855
DDR2	-0.219664022	-0.76065084
DMPK	0.228713494	0.907748695
DSTYK	0.028593664	-0.183224416
DYRK1A	0.3107398	-0.25828098
DYRK1B	0.317143261	0.764849117
DYRK2	0.131801404	-0.126509769
DYRK3	-0.008483575	0.450649922
DYRK4	-0.24045371	-0.194026432
EEF2K	0.128853949	-0.12046606
EGFR	-0.147157071	-1.641781045

EIF2AK1	-0.062384925	-0.071762056
EIF2AK2	-0.084477642	0.085020736
EIF2AK3	-0.016277072	-0.065093025
EIF2AK4	0.029146128	0.205527361
EPHA1	-0.226535552	0.408101429
EPHA10	-0.029804794	0.21944042
EPHA2	0.149706651	0.59803634
EPHA3	0.430752027	0.385285364
EPHA4	-0.234308187	-0.017480968
EPHA5	0.161238689	0.369703254
EPHA6	0.079401773	-0.129084832
EPHA7	0.067430375	0.630194655
EPHA8	0.358513625	0.126234257
EPHB1	0.079743236	0.392987336
EPHB2	-0.241816162	-0.384621599
EPHB3	0.2949426	0.034785702
EPHB4	0.132884422	0.535834511
EPHB6	0.015417363	0.026055464
ERBB2	0.175184952	-0.098620498
ERBB3	0.246664965	-0.056846754
ERBB4	0.071848622	0.032627102
ERN1	0.64945615	0.513930564
ERN2	0.045299616	0.042796972
FASTK	0.004950552	-0.121138386
FER	-0.011152582	0.122229595
FES	-0.113387585	-0.518625918
FGFR1	0.109258656	-0.062233095
FGFR2	-0.017067695	0.059526614
FGFR3	0.18039677	0.336334469
FGFR4	-0.02572632	-0.75538091
FGR	-0.155117593	-0.425894413
FLT1	-0.031153383	0.005184686
FLT3	-0.060209966	-0.786210092
FLT4	0.245157246	0.547651271
FRK	0.083444916	0.030015193
FYN	-0.06445725	-0.158064229
GAK	0.048414194	-0.24015019
GRK1	-0.233247939	-1.065978036
GRK4	-0.277517278	0.243796908
GRK5	-0.053930533	-0.439952534
GRK6	0.150140089	-0.169685792
GRK7	0.118591567	-0.33938197
GSG2	0.128219356	-0.102217171
GSK3A	0.101013358	0.148349021
GSK3B	-0.446535758	-0.240619186
GUCY2C	-0.064315611	-0.112160525
GUCY2D	0.178664991	0.126154234
GUCY2F	-0.068899326	0.532824946
HCK	0.017673969	-0.10408373
HIPK1	-0.148411878	-0.35464774
HIPK2	0.018584527	-0.159993952
HIPK3	0.241633044	0.147021409
HIPK4	0.055609984	-0.202896324
HUNK	0.043629288	0.123888321
ICK	-0.008992935	-0.089361875
IGF1R	-0.049448959	0.233043876
IKBKB	0.190800507	-0.009785969
IKBKE	0.074694569	-0.036853403
ILK	0.058458491	-0.450403348
INSR	-0.062703144	-0.452713945
INSRR	0.246650772	-0.019269712
IRAK1	0.121134426	-0.265300121
IRAK2	0.213246149	0.337815947
IRAK3	0.073381795	-0.145475451
IRAK4	-0.112101554	-0.113792542

ITK	-0.194455978	0.180982511
JAK1	0.020513407	-0.112041314
JAK2	-0.14804715	0.211366437
JAK3	0.471194749	-0.522346323
KALRN	0.09206521	-0.01015583
KDR	0.012992963	-0.212368835
KIT	-0.202356332	-0.546000716
KPNB1	-0.400341246	-1.154955468
KSR1	-0.064080174	0.249374593
KSR2	0.036084064	-0.073037824
LATS1	-0.054417788	-0.081774381
LATS2	-0.117447269	-0.512148693
LCK	-0.198862104	-0.424527962
LIMK1	-0.168244421	-0.041589995
LIMK2	-0.024233273	-0.269221985
LMTK2	-0.15818067	0.12714133
LMTK3	0.498231669	0.549022803
LRRK1	0.230483301	-0.082971458
LRRK2	0.017210393	-0.141683008
LTK	0.067802381	0.379303679
LYN	-0.103077229	-0.249238154
MAK	0.045065275	0.176255874
MAP2K1	0.135257586	-0.109978243
MAP2K2	-0.152551528	0.075192952
MAP2K3	-0.144195201	-0.397645872
MAP2K4	0.024089808	0.142661753
MAP2K5	0.285471807	0.83240899
MAP2K6	0.162141	0.344998642
MAP2K7	0.151803277	0.12170712
MAP3K1	-0.124628955	-0.030826047
MAP3K10	0.274078525	0.477211555
MAP3K11	0.073459819	-0.363292005
MAP3K12	-0.099345796	-0.442792492
MAP3K13	-0.122429356	-0.448735639
MAP3K14	-0.120766443	0.033210025
MAP3K15	-0.063632088	-0.00811727
MAP3K19	0.055842668	0.083341499
MAP3K2	0.123588413	-0.306227073
MAP3K3	0.580398084	0.464548324
MAP3K4	-0.043943498	0.230827929
MAP3K5	0.07999745	-0.475336604
MAP3K6	0.278686124	-0.307111815
MAP3K7	-0.403802038	-0.49371681
MAP3K8	-0.033196812	0.367547011
MAP3K9	0.023047742	0.461624876
MAP4K1	0.163379558	0.419423433
MAP4K2	-0.01599484	-0.23023328
MAP4K3	0.133174027	0.195082755
MAP4K4	-0.03286409	-0.672420089
MAP4K5	0.092842181	0.124769301
MAPK1	0.187420814	-0.897487556
MAPK10	0.08240118	-0.2350895
MAPK11	0.114249327	-0.154118953
MAPK12	0.138476758	0.043184838
MAPK13	0.127704551	-0.29817815
MAPK14	0.438260786	0.33159921
MAPK15	-0.150874251	-0.20641477
MAPK3	0.248861055	0.299625591
MAPK4	0.215674552	0.162727003
MAPK6	0.497813937	0.284757308
MAPK7	0.022575628	-0.412751241
MAPK8	0.115024295	-0.228662174
MAPK9	0.233346954	0.42323917
MAPKAPK2	-0.010305403	-0.245575517
MAPKAPK3	0.165053363	0.45804366

MAPKAPK5	-0.058247416	0.126765479
MARK1	-0.071908569	-0.315037017
MARK2	0.011364761	-0.059642921
MARK3	-0.140979941	-0.258301424
MARK4	0.067495472	0.012134627
MAST1	0.08833259	0.172513311
MAST2	0.103375117	0.437383747
MAST3	0.071823133	-0.082385664
MAST4	0.045892709	-0.258243729
MASTL	-0.212196214	-1.341092252
MATK	0.21229065	-0.729682141
MELK	0.074685508	0.114728756
MERTK	-0.058205666	-0.314195818
MET	0.048419206	-0.406651637
MINK1	-0.062395538	0.000679064
MKNK1	-0.237291263	-0.017731708
MKNK2	-0.069776167	0.031492242
MLKL	-0.005462082	-0.031751102
MOK	0.079779494	0.012064185
MOS	0.309870168	-0.117863021
MST1R	-0.09090131	-0.554855077
MST4	0.138099793	0.459156684
MTOR	0.050976219	-0.108726205
MUSK	-0.051115281	-0.190388524
MYLK	-0.081591917	0.257339179
MYLK2	-0.178126579	-0.07121361
MYLK3	-0.147860881	-0.651625947
MYLK4	0.443730165	0.546959727
MYO3A	0.327101201	0.731429181
MYO3B	0.106045742	-0.282180087
NEK1	-0.027333656	0.013779469
NEK10	-0.151773368	-0.229619133
NEK11	0.040188818	0.315339851
NEK2	-0.010897253	-0.240488438
NEK3	-0.05317017	0.15763533
NEK4	-0.075027687	-0.537496512
NEK5	0.091861096	0.085712489
NEK6	-0.006415776	0.07960182
NEK7	-0.091640272	-0.280856906
NEK8	-0.084991417	0.214093814
NEK9	-0.218028763	0.17481302
NIM1	0.221429591	0.915405546
NLK	0.012658943	0.134170398
NPR1	0.61698953	0.754518362
NPR2	0.057808935	0.232307735
NRBP1	0.102973999	-0.162247111
NRBP2	0.15272422	0.124394738
NRK	-0.051027353	0.505217986
NTRK1	0.162594459	-0.523632819
NTRK2	0.254764676	0.34933193
NTRK3	0.200665019	-0.579743196
NUAK1	-0.27983059	-0.908567946
NUAK2	-0.038372529	-0.32214565
NUP98	-0.147507972	-0.440889813
OBSCN	0.245128835	0.446415545
OXSR1	-0.026292783	-0.070714748
PAK1	-0.067935796	-0.224177545
PAK2	0.133490114	0.289210484
PAK3	0.03992829	-0.023960838
PAK4	0.43990776	0.369808506
PAK6	0.003886527	0.235592313
PAK7	0.117829589	-0.244057856
PAN3	-0.229206958	-0.113195678
PASK	0.105066751	0.170189047
PBK	-0.41043652	-0.162408363

PDGFRA	-0.089457891	-0.310119791
PDGFRB	0.209509208	0.147444576
PDIK1L	-0.104243328	-0.719458171
PDK1	0.056290467	-0.127079194
PDK2	0.07317829	0.184483844
PDK3	-0.071785036	-0.14578722
PDK4	0.167991023	0.852777036
PDPK1	0.184910542	0.183211601
PEAK1	0.085376851	0.255280431
PHKG1	0.378828132	0.60684033
PHKG2	0.64253281	0.498204472
PIK3CA	-0.084649462	-0.643807647
PIK3CG	0.513162218	0.827481024
PIK3R4	0.004730107	0.16946665
PIM1	-0.10100194	-0.35035874
PIM2	0.056129594	0.007482934
PIM3	0.213179717	-0.442883171
PINK1	-0.10000958	0.197287778
PKDCC	0.063220305	-0.097493153
PKMYT1	-0.184376387	-0.415074735
PKN1	0.086256813	0.14301965
PKN2	-0.013764419	-0.154918402
PKN3	-0.040799969	0.081612454
PLK1	-0.308439965	-1.545514996
PLK2	0.223568141	-0.290602426
PLK3	-0.471327306	0.021914102
PLK4	-0.299805914	-0.455915778
PLK5	-0.27719719	-0.580654265
PNCK	0.127936079	0.162861193
PRKAA1	-0.32285181	-0.70717916
PRKAA2	-0.029432214	-0.723689312
PRKACA	0.180058754	-0.049216278
PRKACB	-0.025654037	-0.09987617
PRKACG	0.05080494	-0.094883478
PRKCA	-0.152364017	-0.256524517
PRKCB	0.009301069	-0.208792018
PRKCD	0.403104278	-0.219057275
PRKCE	0.027585732	0.107929256
PRKCG	0.174948867	0.119653673
PRKCH	0.211581706	-0.00462546
PRKCI	-0.071851074	-0.410516841
PRKCQ	0.096475813	0.001927329
PRKCZ	-0.031551814	0.001620596
PRKD1	0.423393082	0.92801936
PRKD2	0.04966237	0.336686477
PRKD3	-0.100820027	-0.740844724
PRKDC	0.08067562	-0.864783043
PRKG1	0.220935341	-0.151978827
PRKG2	-0.30020559	-0.25386807
PRKX	-0.022043078	-0.031837352
PRPF4B	0.028630329	-0.272738426
PSKH1	0.544963952	0.468901888
PSKH2	0.68872349	0.788754639
PSMB2	-0.38620384	-1.389305873
PSMC4	-0.200479513	-0.881583806
PSMD11	-0.272597047	-1.454092618
PSMD6	0.162413248	-0.370387136
PTK2	-0.315951666	-0.135482822
PTK2B	0.118968594	0.447163992
PTK6	0.048309919	-0.406526403
PTK7	0.070688617	0.130976067
PXK	-0.44763616	0.088945308
RAF1	0.025662174	-0.52051626
RET	-0.026599613	0.176594653
RIOK1	-0.12506712	-0.13906053

RIOK2	-0.10065487	-0.604293105
RIOK3	-0.13478859	-0.752011979
RIPK1	-0.185942839	-0.267098824
RIPK2	0.182080871	0.864410832
RIPK3	0.300785651	-0.411645765
RIPK4	0.234562771	-0.235209974
RNASEL	0.032250472	-0.198038716
ROCK1	-0.078997261	-0.542224068
ROCK2	0.327549943	0.447394493
ROR1	0.022552083	0.358922789
ROR2	-0.096788927	-0.290687723
ROS1	-0.024813347	0.106004902
RPL11	-0.214228182	-1.441082822
RPL3	0.118902438	-0.511096286
RPS13	-0.392743437	-1.366497452
RPS6KA1	0.435145553	0.816494155
RPS6KA2	0.285885606	0.37657522
RPS6KA3	0.020672533	0.244369114
RPS6KA4	-0.141287426	-0.868075037
RPS6KA5	0.000464157	-0.184214262
RPS6KA6	-0.147614848	0.004489321
RPS6KB1	0.038766535	0.273968288
RPS6KB2	-0.053918717	-0.025437232
RPS6KC1	0.113748401	0.235500535
RPS6KL1	-0.145349396	-1.027846247
RYK	0.580368501	0.450713786
SBK1	0.172333055	0.11057052
SBK2	0.438706146	0.250328024
SCYL1	0.114015245	-0.666382252
SCYL2	0.093013078	-0.003736451
SCYL3	-0.17125344	-0.018229802
SGK1	0.029432089	0.262230653
SGK2	0.241179543	0.295778878
SGK223	-0.043506385	0.130390203
SGK3	-0.42622243	0.356356471
SGK494	-0.23338116	-0.543499192
SIK1	0.093854439	0.030031735
SIK2	0.058871339	-0.592506823
SIK3	-0.073520056	-0.082066649
SLK	0.095116376	0.565010413
SMG1	-0.106808247	-0.49147156
SNRK	0.045668473	0.102884964
SPEG	0.033399607	0.255397351
SRC	-0.0796321	-0.672452224
SRMS	-0.010374199	-0.239526742
SRPK1	-0.174236705	-0.243719179
SRPK2	0.409679501	0.256866458
SRPK3	0.021561215	0.16767045
STK10	-0.093620124	-0.093655304
STK11	-0.019965911	-0.075381173
STK16	-0.116853508	-0.50574359
STK17A	-0.054623515	-0.194027977
STK17B	0.280418474	0.674365658
STK24	0.00326119	0.059928152
STK25	-0.13930643	-0.258999755
STK3	0.157213517	0.140638959
STK31	0.112190483	0.417512708
STK32A	0.273741242	0.646143354
STK32B	-0.069862866	-0.409120857
STK32C	0.106023671	0.344503182
STK33	0.06404187	1.009645397
STK35	-0.141385123	-0.271012813
STK36	-0.205650476	-0.952987409
STK38	-0.310023665	-0.37741552
STK38L	-0.04138906	-0.130732768

STK39	-0.045425316	-0.004353929
STK4	0.284816971	0.738566449
STK40	0.010582295	-0.163146584
STRADA	-0.030610287	-0.676619112
STRADB	-0.002137982	0.316723463
STYK1	0.411120293	1.334045076
SYK	0.010643757	0.309270375
TAOK1	0.065890946	-0.152614015
TAOK2	-0.281212951	-0.036801427
TAOK3	0.138628768	0.239170336
TBCK	0.254940076	0.090309899
TBK1	-0.090472631	-0.496911103
TEC	-0.255284976	0.077248535
TEK	-0.211856032	-0.485004068
TESK1	-0.167076092	-0.076928398
TESK2	-0.21467638	-0.23871552
TEX14	-0.168168458	0.060012132
TGFBR1	0.130325687	0.400778643
TGFBR2	0.229224407	0.327772737
TIE1	-0.026625908	-0.130166242
TLK1	-0.286474755	-0.493237008
TLK2	0.138127736	-0.682762454
TNIK	0.050556799	0.603492067
TNK1	0.212036237	0.413166296
TNK2	0.072750533	-0.145995167
TNNI3K	-0.094839371	-0.389710366
TP53RK	0.333991347	-0.095851241
TRIB1	0.237700211	-0.098921046
TRIB2	-0.115610423	-0.085372069
TRIB3	-0.164515408	-0.213736884
TRIO	-0.030309064	-0.282775995
TRPM6	0.438585131	0.317120389
TRPM7	0.012231325	0.365872451
TSSK1B	-0.082691604	0.192770071
TSSK2	-0.414284586	-0.111307042
TSSK3	0.131797185	-0.267804196
TSSK4	-0.239303177	-0.356270807
TSSK6	0.077982005	-0.403890925
TTBK1	0.247025942	0.414760285
TTBK2	-0.017478223	-0.005139454
TTK	-0.276094015	-0.792258868
TTN	0.204214376	0.396860458
TXK	0.609037665	-0.918237897
TYK2	0.0430755	0.241680183
TYRO3	-0.178016959	-0.778635188
UHMK1	0.188676969	0.324688001
ULK1	0.224147659	0.731923481
ULK2	0.043338765	-0.058137008
ULK3	-0.007415541	0.075165116
ULK4	0.05677821	0.461007369
VRK1	-0.094365738	-0.122805443
VRK2	0.008071645	-0.056569074
VRK3	-0.26459924	0.052034629
WEE1	-0.224624254	-0.985821912
WEE2	0.303317634	0.049687945
WNK1	0.238614533	0.286471795
WNK2	-0.071820315	-0.423172002
WNK3	0.069783777	0.828334779
WNK4	0.325540601	0.733034795
YES1	-0.171436957	-0.425372904
ZAK	-0.064568888	0.401644154
ZAP70	0.143563785	0.029526631
NonTargetingControlGuideForHuman_0050	0.286100393	0.279114611
NonTargetingControlGuideForHuman_0049	1.025644531	0.999188037
NonTargetingControlGuideForHuman_0048	0.504376705	0.716079949

NonTargetingControlGuideForHuman_0047	0.434835772	1.237463075
NonTargetingControlGuideForHuman_0046	0.072093214	0.371040726
NonTargetingControlGuideForHuman_0045	0.286100393	1.183195065
NonTargetingControlGuideForHuman_0044	0.39945621	-0.438873011
NonTargetingControlGuideForHuman_0043	-0.08292301	0.515149327
NonTargetingControlGuideForHuman_0042	1.734052514	4.706865727
NonTargetingControlGuideForHuman_0041	0.440748207	1.257562621
NonTargetingControlGuideForHuman_0040	0.18895791	1.61169655
NonTargetingControlGuideForHuman_0039	0.364049318	0.163029137
NonTargetingControlGuideForHuman_0038	0.516163827	1.484654106
NonTargetingControlGuideForHuman_0037	-0.403676343	0.367327282
NonTargetingControlGuideForHuman_0036	-0.35508274	1.036620983
NonTargetingControlGuideForHuman_0035	0.150654759	0.776151923
NonTargetingControlGuideForHuman_0034	-0.075693836	1.358474092
NonTargetingControlGuideForHuman_0033	-0.367977268	-0.24922383
NonTargetingControlGuideForHuman_0032	0.051324247	1.351889216
NonTargetingControlGuideForHuman_0031	0.701137892	1.245584987
NonTargetingControlGuideForHuman_0030	0.169792369	0.575499532
NonTargetingControlGuideForHuman_0029	0.33330935	0.825414971
NonTargetingControlGuideForHuman_0028	-0.410503389	0.945428749
NonTargetingControlGuideForHuman_0027	0.362641947	0.606025037
NonTargetingControlGuideForHuman_0026	-0.126523829	-0.476868695
NonTargetingControlGuideForHuman_0025	1.030445517	1.022214083
NonTargetingControlGuideForHuman_0024	0.196476602	-0.674980545
NonTargetingControlGuideForHuman_0023	0.357988211	0.766185315
NonTargetingControlGuideForHuman_0022	-0.162578052	-0.497791487
NonTargetingControlGuideForHuman_0021	0.226882174	-0.729365317
NonTargetingControlGuideForHuman_0020	-0.555322284	-0.217897734
NonTargetingControlGuideForHuman_0019	0.942354187	1.205404311
NonTargetingControlGuideForHuman_0018	-0.269222618	-0.589301837
NonTargetingControlGuideForHuman_0017	0.112907677	0.604103749
NonTargetingControlGuideForHuman_0016	-0.539870208	-0.045815972
NonTargetingControlGuideForHuman_0015	-0.249090811	0.66543834
NonTargetingControlGuideForHuman_0014	-1.117946323	-0.750324936
NonTargetingControlGuideForHuman_0013	-0.046269449	0.112743227
NonTargetingControlGuideForHuman_0012	0.029854854	0.243714132
NonTargetingControlGuideForHuman_0011	-0.157595399	0.437436484
NonTargetingControlGuideForHuman_0010	0.913406096	1.650245305
NonTargetingControlGuideForHuman_0009	0.019261779	-0.150539555
NonTargetingControlGuideForHuman_0008	-0.254343441	0.38193917
NonTargetingControlGuideForHuman_0007	-0.084670168	0.242620307
NonTargetingControlGuideForHuman_0006	-0.013609663	0.663612529
NonTargetingControlGuideForHuman_0005	0.239392407	0.880291299
NonTargetingControlGuideForHuman_0004	0.106766477	0.339031608
NonTargetingControlGuideForHuman_0003	-0.421080903	0.042675162
NonTargetingControlGuideForHuman_0002	0.369408984	0.082169657
NonTargetingControlGuideForHuman_0001	0.1656051	0.29992191

Table S2. Primers for PCR assays

RT-qPCR primer sequences	
Primer labels	Sequences
Primer lables	Sequences
EFFRI1 #F	CTACTGGAGCAGTCGCAGTG
EFFRI1 #R	CCTCTTCATGTGGTCCCAAG
β-Actin #F	GGGACCTGACTGACTACCTC
β-Actin #R	ATCTTCATTGTGCTGGGTG

siRNA sequences	
Target genes	Sequences
human *EFFRI1* si1	CGAUAAUAGAACUAGUGACTT
human *EFFRI1* si2	GCUAUGUGUCUGACCAAAATT
human *HRAS* si1	AGAGGATTCCTACCGGAAGCA
human *HRAS* si2	CGGAAGCAGGTGGTCATTGAT
human *NRAS* si1	GAAACCTGTTTGTTGGACATA
human *NRAS* si2	CAGTGCCATGAGAGACCAATA
human *MRAS* si1	CCACCTCTCAATGTCGACAAA
human *MRAS* si2	CCTGAAACATACGGAGATTGA

shRNA sequences	
Target genes	Sequences
human *EGFR* sh1	CCGGCCTCCAGAGGATGTTCAATAACTCGA
	GTTATTGAACATCCTCTGGAGGTTTTTG
human *EGFR* sh2	CCGGGCCACAAAGCAGTGAATTTATCTCGA
	GATAAATTCACTGCTTTGTGGCTTTTTG

Table S3. Body weight of *KRAS*^{G12D}-mutant CRC cell xenograft mice

LS180 xenograft mouse model				
Item	Group			
	Vehicle	MRTX1133	Cetuximab	Combo
Day 0	22.8±1.3	22.2±2.2	22.9±1.4	23.5±1.5
Day 3	23.8±1.8	22.74±2.6	24.1±1.8	23.6±1.8
Day 6	23.5±1.5	22.6±2.1	23.9±1.9	23.3±1.7
Day 9	24.1±1.3	24.5±1.8	23.6±1.7	22.1±1.6
Day 12	23.9±1.1	23.5±2.1	24.1±1.7	23.3±1.6
Day 15	23.9±1.2	20.7±2.4	23.4±1.8	23.0±1.4
Day 18	25.7±1.4	20.9±2.2	24.7±1.7	24.4±1.6
Day 21	25.8±1.9	22.2±3.9	25.1±2.2	24.6±1.7

LS174T xenograft mouse model				
Item	Group			
	Vehicle	MRTX1133	Cetuximab	Combo
Day 0	23.5±1.6	22.3±1.5	22.9±1.1	24.2±1.0
Day 3	24.3±2.4	25.0±1.4	24.9±0.8	24.9±1.8
Day 6	24.3±2.4	25.0±1.6	24.8±0.9	25.1±1.9
Day 9	24.5±2.7	23.1±1.3	24.5±1.1	25.0±1.2
Day 12	23.6±1.7	23.4±1.2	23.5±0.9	24.1±1.2
Day 15	23.0±1.8	21.8±0.9	22.4±1.1	22.4±1.0
Day 18	24.5±2.3	22.2±0.9	22.7±1.1	22.6±1.3
Day 21	25.0±1.7	22.1±1.4	22.7±0.8	22.7±0.8

Table S4. Biochemistry testing of plasma from *KRAS*^{G12D}-mutant CRC cell xenograft mice

LS180 xenograft mouse model			
Item	Group	Value	P-value (Treatment vs.
CR (μmol/L)	Vehicle	15.80±2.05	
	MRTX1133	13.60±2.30	>0.05
	Cetuximab	13.80±1.30	>0.05
	Combo	13.00±1.41	>0.05
BUN (mmol/L)	Vehicle	7.62±1.23	
	MRTX1133	9.05±1.43	>0.05
	Cetuximab	8.36±0.66	>0.05
	Combo	8.08±0.73	>0.05
AST/ALT	Vehicle	5.00±0.30	
	MRTX1133	3.78±0.74	<0.05
	Cetuximab	3.93±0.69	<0.01
	Combo	4.19±0.30	<0.01

LS174T xenograft mouse model			
Item	Group	Value	P-value (Treatment vs.
CR (μmol/L)	Vehicle	15.20±1.20	
	MRTX1133	14.50±2.06	>0.05
	Cetuximab	15.4±1.14	>0.05
	Combo	14.6±1.67	>0.05
BUN (mmol/L)	Vehicle	6.89±0.60	
	MRTX1133	7.60±0.51	>0.05
	Cetuximab	8.28±0.89	>0.05
	Combo	9.37±1.26	<0.01
AST/ALT	Vehicle	3.09±1.17	
	MRTX1133	2.61±1.33	>0.05
	Cetuximab	3.87±091	>0.05
	Combo	2.62±0.53	>0.05
